# Vital signs and common blood tests improve the predictive power of the Hospital Frailty Risk Score to predict poor outcomes across all adult ages

**DOI:** 10.1371/journal.pone.0348669

**Published:** 2026-05-05

**Authors:** Huda Kutrani, Jim Briggs, David Prytherch, Claire Spice

**Affiliations:** 1 Centre for Healthcare Modelling and Informatics, University of Portsmouth, Portsmouth, United Kingdom; 2 Faculty of Public Health, University of Benghazi, Benghazi, Libya; 3 Queen Alexandra Hospital, Portsmouth Hospitals University Trust, Portsmouth, United Kingdom; Japanese Red Cross Medical Center, JAPAN

## Abstract

**Background:**

Frailty is associated with poor health outcomes and is a public health challenge worldwide. The Hospital Frailty Risk Score (HFRS) has been widely used to identify patients at risk of frailty and predict poor outcomes including long length of stay (LOS) and in-hospital mortality for older patients. This study aimed to explore and determine variables that might influence the ability of the Hospital Frailty Risk Score to predict LOS and in-hospital mortality across all adult ages.

**Methods:**

This is a retrospective cohort study using data from Queen Alexandra Hospital in Portsmouth, UK of consecutive patient admissions over 10 years between 01/01/2010 to 31/12/2019. The study included patients aged 16 years and older. The HFRS was calculated for each patient based on ICD-10 diagnostic codes with a 2-year look-back. The National Early Warning Score (NEWS) and the Laboratory Decision Tree Early Warning Score (LDT-EWS) were calculated for each patient. Vital signs and blood tests were the first available routine data from patients after admission. We developed logistic regression models (alone and adjusted) for 9 prediction periods of length of stay and 8 prediction periods of in-hospital mortality and assessed the model performance using AUROC.

**Results:**

Combining HFRS with the LDT-EWS had the highest discrimination (AUROC ranging from 0.764 to 0.810) compared to adjusted models (AUROC ranging from 0.716 to 0.796) or HFRS alone (AUROC ranging from 0.723 to 0.798) for 9 periods of length of stay. For in-hospital mortality, combining HFRS with NEWS had the highest discrimination (AUROC ranging from 0.786 to 0.829) compared to HFRS alone or HFRS combined with other variables for 3, 7, 10 and 14-day mortality across all adult ages. And combining HFRS with LDT-EWS had the highest discrimination (AUROC ranging from 0.789 to 0.794) for mortality after more than 14 days across all adult ages.

**Conclusions:**

Combining HFRS with additional routinely available variables significantly improves the predictive power for length of stay and mortality. This is the first paper to show that LDT-EWS significantly improves the predictive power of Hospital Frailty Risk Scores to predict longer length of stay in hospital and later in-patient mortality across all adult ages. The predictive power of the HFRS was improved by NEWS for early in-patient mortality.

## Introduction

Frailty is a medical syndrome characterized by reduced physiological function and increased vulnerability to stressors such as hospital admission. It is associated with morbidity and increased risk of poor health outcomes and is a reliable measure to predict health outcomes such as length of stay and mortality [1–3]. Identifying patients at high risk of frailty assists in enabling timely interventions that reduce the risk of poor health outcomes, reduce healthcare resource use, and improve patient care [1–4]. However, identification of those with frailty is not routine in many systems and where it is mandated, such as in the English National Health System, take up is not universal [5].

Although frailty increases with age, it is also prevalent in younger adults [6–8] and has been identified as a strong predictor of length of stay (LOS) and mortality in adults of all ages [8–10]. Tools to measure frailty are becoming increasingly important in identifying people at a high risk of poor outcomes but many require face-to-face assessment, which can be difficult in an acute care setting [11–13]. The Hospital Frailty Risk Score (HFRS) was developed initially in older people in hospital and has the advantage of being based on ICD 10 diagnosis codes [11,14]. Subsequently it has since been validated in various countries and in speciﬁc populations to identify older people at risk of frailty and predict longer LOS and in-hospital mortality [12–16]. The recent studies found that HFRS can predict hospital LOS [17] and in-hospital mortality [18] across all adult ages, not just for older people. It can be implemented in most hospital information systems at low cost and without additional burden on clinical staff [11,12,14,19].

Whilst frailty scoring and scales can identify those at risk of longer hospital stays and mortality there is potential to improve prediction through combination with other relevant variables. Further evaluation of the HFRS in combination with variables such as physiological scores and in more differentiated cohorts has been suggested as a focus for research [20].The majority of studies have included patients’ characteristics such as age, sex, and Charlson Comorbidity Index (CCI) with HFRS to predict long hospital stay and in-hospital mortality and the predictive power of the HFRS models improved [11,16,21] or remained significant [12–14]. However, common blood tests and vital signs, along with age and gender are also predictors of poor health outcomes [22–26]. Vital signs represented by NEWS [23] and 7 common blood tests (Haemoglobin, Urea, Potassium, Sodium, White Cell Count, Creatinine, and Albumin) represented by LDT EWS (Laboratory Decision Tree Early Warning Score) [22] can assist in the detection of clinical deterioration in patients. Previous studies showed that NEWS [23,24,27,28], LDT-EWS [22] and separate blood tests (a part of LDT-EWS) [22,26,29,30] are predictive of poor outcomes. Some studies reported that adding blood tests results [31,32] and vital signs [27,28] to prediction models improved the predictive power of the models. Adding a clinical frailty scale to a variety of physiological scores has been shown to improve the prediction of admission to hospital from the Emergency Department and in-hospital mortality in older people [33].

The analysis of factors that may influence the prediction of HFRS for poor outcomes, including readily available clinical data, has the potential to improve the ability to identify those who are admitted to the hospital who are at risk of a prolonged length of stay or in-hospital mortality. Therefore, this study aimed to explore whether other variables such as laboratory results, physiological scoring and comorbidity influence the predictive power of HFRS to predict length of stay and in-hospital mortality across all adult ages.

## Methods

### Study design and participants

This study follows similar methods to those of Kutrani et al. (2025) and summarizes them here for any convenience [17,18,34]. This is a retrospective cohort study of non-elective patients who were admitted to a large acute hospital (Queen Alexandra Hospital in Portsmouth, UK) from 1st January 2010–31st December 2019. The study included patients aged 16 years and older who had not registered for the national data opt-out to stop their data being used for research.

**Fig 1:**
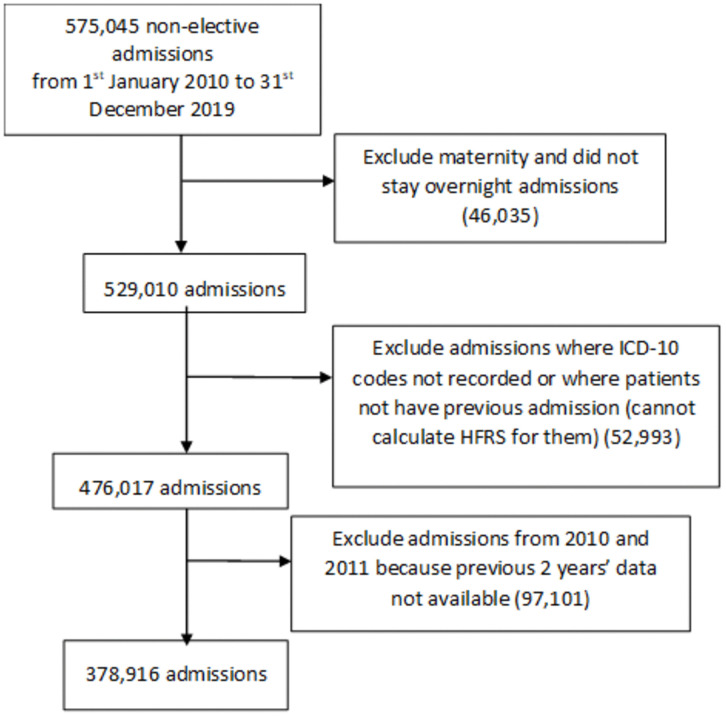
Flowchart showing the inclusion and exclusion criteria for the study.

The full dataset was 575,045 non-elective admissions. Since the calculation of HFRS relies on having 2 previous years’ data for optimal construction of HFRS [14], the analysis was based on patients admitted from 1^st^ January 2012–31^st^ December 2019. We excluded maternity cases and certain types of admission from the study (eligible admissions were 378,916 admissions) as described in Fig 1.

### Hospital frailty risk score calculation

The HFRS was calculated for each patient, based on 109 International Classification of Diseases 10th revision (ICD-10) diagnoses documented in their hospital admission records. The final HFRS for each patient is calculated by adding the weighted points together for each code present from the index admission with any diagnosis recorded from previous admissions during the previous two years [11,14]. In addition, HFRS were categorised into low frailty risk (score less than 5), intermediate frailty risk (5–15), and high frailty risk (>15) as per the original study [11].

### Outcomes and variables

We determined the predictive ability of the HFRS on nine periods of length of stay and eight periods of in-hospital mortality (death discharge) as outcomes of the study. These prediction periods were shown in Table 1.

**Table 1:** Prediction periods of length of stay and in-hospital mortality.

9 periods of LOS	8 periods of in-hospital mortality
LOS > 3 days	3-day mortality
LOS > 7 days	7-day mortality
LOS > 10 days	10-day mortality
LOS > 14 days	14-day mortality
LOS > 21 days	30-day mortality
LOS > 30 days	60-day mortality
LOS > 45 days	90-day mortality
LOS > 60 days	6-month mortality
LOS > 90 days	

Variables were HFRS, age, gender, Charlson Comorbidity Index (CCI), aggregate NEWS, c-reactive protein test (CRP), and aggregate LDT-EWS.

Laboratory tests and vital signs studied were the first available results gathered from patients at the point of the hospital stay. Most of the blood tests and vital signs were collected on the first day of admission. The seven vital signs were Respiration Rate (RR), body temperature (Temp), Systolic Blood Pressure (SBP), Saturation of Peripheral Oxygen (SPO2), Heart Rate (HR), ACVPU, and Oxygen state. The NEWS is an aggregate score that represents these vital signs, with a higher score indicating increased risk of poor outcomes [23].

The LDT-EWS is based on seven blood tests: Haemoglobin (HB), Urine (U), Potassium (K), Sodium (Na), White Cell Count (WCC), Creatinine (CR), and Albumin (Alb). For each test, LDT-EWS assigns a score from 0 to 3, which are then summed to give an aggregate score under one variable named LDT_EWS. A higher value of LDT-EWS represents a higher relative risk of in-hospital death [22].

We included C-reactive protein (CRP) separately because it is not included in LDT-EWS. An abnormal level of CRP is typically associated with increased risk of longer LOS and in-hospital mortality [35]. An elevated CRP signifies infection or inflammation. The CRP ranges from <1 mg/L to over 500 mg/L. A value below 3.0 mg/L is normal; a value from 3.0–10.0 mg/L is slightly elevated. A value from 10.0 to 100.0 mg/L is moderately elevated, 100.0 to 500.0 mg/L is elevated, and above 500 mg/L is severely elevated [36].

### Statistical analysis

Patient characteristics were described using mean, standard deviation, median, and interquartile range (IQR) for continuous variables, and number and percentage for categorical variables.

Logistic regression models were developed with nine LOS and eight in-hospital mortality periods as outcomes. In the first group, models used each variable alone, including the HFRS score to predict each period of LOS and in-hospital mortality. In the second group, models used the HFRS score (a continuous variable) in combination with one other variable to predict each outcome of LOS and in-hospital mortality. In the third group a multivariate model (including all variables in our study) was used. For LOS analysis, we excluded patients who died in the hospital, because patients who died in the hospital had a shorter length of stay than if they had survived, which may have a negative effect on predicting longer LOS [37]. Further LOS analysis was performed for all patients, including those who died in the hospital. The study cohort was randomly split into 70% training dataset and 30% validation dataset.

The predictive power of models was assessed by discrimination using the Area Under Receiver Operating Characteristic (AUROC or c-statistic) with 95% confidence intervals (95% CI). A value of AUROC below 0.6 indicates no discrimination; values ranging from 0.60–0.7 indicate poor discrimination, values of 0.7–0.8 indicate fair discrimination, values from 0.8–0.9 indicate good discrimination, and values above 0.9 indicate excellent discrimination [38]. We compared the AUROC value of the HFRS alone model with other models for each period of LOS and in-hospital mortality. A higher AUROC indicated improved predictive power of the model. P values <0.05 were considered statistically significant. Data manipulation and logistic regression modelling were performed using R-Studio version 4.2.1.

### Cross validation

We conducted validation experiments to evaluate the stability of the models to see whether the discrimination (AUROC) of the best model is the best predictor for the nine periods of LOS and eight periods of in-hospital mortality. We evaluated:

- What is the best model for each LOS and in-hospital mortality period?
- Do different samples of training and testing datasets have different results?
- Does the best model always consistently give the best results for any sample data?

In addition, we generated several samples of data for validation experiments which included the following:

- 8 samples of data according to admission year – each calendar year from 2012 to 2019.
- 4 samples of data according to age (<45 years, 45–64 years, 65–84 years, and ≥85 years).
- 2 samples of data according to gender.

Full details of these samples are reported in S1 Table.

### Ethics

The study included patients aged 16 years and older who had not registered for the national data opt-out for their data being used for research. We accessed data from the Portsmouth CORE-D routine care data repository on 1st November 2022. The research team did not have access to any information that could identify individual patients during or after data collection.

The dataset used in this study was covered by existing ethical approval granted by an NHS Research Ethics Committee in April 2021. REC reference is 21/SC/0080. IRAS project ID is 281193. The data was made available by Portsmouth Hospital University NHS Trust under a data sharing agreement.

## Results

A total of 378,916 non-elective admissions for patients aged 16 years and older were included. Patients had HFRS ranging from 0 to 77 with a mean of 7.1. Regarding the HFRS category, 223414 (59.0%) of 378916 admissions were categorised as low risk of frailty, 96623 (25.5%) of 378916 admissions were categorised as intermediate risk of frailty, and 58879 (15.5%) of 378916 admissions were categorised as high risk of frailty. The mean age was 61.8 years and 54.9% were females. The mean and median aggregate LDT-EWS was 2.0 and 1, NEWS was 1.9 and 1, CCI was 4.8 and 0, and CRP was 49.2 and 13.0. For about 40% of admissions, the speciality of discharge was General Medicine, followed by 12.2% for Accident and Emergency (S2 Table).

### HFRS and length of stay prediction

Patients’ characteristics in the dataset and each of the length of stay period subsets are presented in Table 2. Mean HFRS increased with a longer length of stay. The mean and median aggregate LDT-EWS was around 3.2 in all periods of length of stay. The mean and median aggregate NEWS were low risk in all length of stay periods. In general, CCI increased with the length of stay but then decreased after (LOS > 21 days). The mean and median CRP level was “moderately elevated” in all length of stay periods.

**Table 2:** Characteristics of patients by predicted length of stay periods.

	prediction Length of Stay (LOS)								
	LOS > 3days N = 128,606	LOS > 7days N = 74,677	LOS > 10days N = 54,948	LOS > 14days N = 38,955	LOS > 21days N = 23,497	LOS > 30days N = 14,020	LOS > 45days N = 6,783	LOS > 60days N = 3,484	LOS > 90days N = 1,059
**HFRS**									
Mean (SD)	10.8 (10.7)	12.8 (11.2)	13.7 (11.3)	14.5 (11.4)	15.4 (11.4)	15.9 (11.4)	16.2 (11.3)	16.2 (11.2)	16.2 (10.2)
Median (IQR)	7.4 (2.6–15.7)	10.0 (4.2–18.6)	11.0 (5.1–19.6)	12.0 (5.8–20.4)	13.0 (6.6–21.6)	13.6 (7.1–22.2)	14.0 (7.6–22.4)	14.0 (7.9–22.1)	14.1 (8.6–21.6)
**Age in years**									
Mean (SD)	71.6 (17.9)	75.0 (16.0)	76.3 (15.5)	77.0 (14.9)	77.5 (14.8)	77.2 (14.9)	76.6 (14.8)	75.2 (15.3)	72.4 (16.2)
Median (IQR)	76.0 (62.0–85.0)	79.0 (67.0–87.0)	80.0 (69.0–87.0)	81.0 (70.0–88.0)	81.0 (71.0–88.0)	81.0 (70.0–88.0)	81.0 (69.0–87.0)	79.0 (67.0–86.0)	76.0 (63.0–85.0)
**LDT-EWS**									
Mean (SD)	2.9 (2.4)	3.2 (2.4)	3.3 (2.4)	3.3 (2.4)	3.3 (2.4)	3.2 (2.3)	3.2 (2.3)	3.2 (2.3)	3.2 (2.3)
Median (IQR)	2.0 (1.0–4.0)	3.0 (1.0–4.0)	3.0 (2.0–4.0)	3.0 (2.0–4.0)	3.0 (2.0–4.0)	3.0 (1.0–4.0)	3.0 (1.0–4.0)	3.0 (1.0–4.0)	3.0 (1.0–4.0)
**NEWS**									
Mean (SD)	2.3 (2.4)	2.4 (2.4)	2.4 (2.4)	2.5 (2.4)	2.5 (2.4)	2.5 (2.4)	2.4 (2.3)	2.4 (2.3)	2.4 (2.3)
Median (IQR)	2.0 (0.0–3.0)	2.0 (0.0–4.0)	2.0 (1.0–4.0)	2.0 (1.0–4.0)	2.0 (1.0–4.0)	2.0 (1.0–4.0)	2.0 (0.0–4.0)	2.0 (0.0–4.0)	2.0 (0.0–4.0)
**CCI**									
Mean (SD)	7.1 (10.5)	7.9 (11.1)	8.2 (11.3)	8.3 (11.4)	8.3 (11.3)	7.8 (11.1)	7.2 (10.7)	6.6 (10.2)	5.7 (9.6)
Median (IQR)	0.0 (0.0–12.0)	3.0 (0.0–14.0)	3.0 (0.0–14.0)	3.0 (0.0–14.0)	3.0 (0.0–14.0)	0.0 (0.0–14.0)	0.0 (0.0–13.0)	0.0 (0.0–11.0)	0.0 (0.0–10.0)
**CRP**									
Mean (SD)	68.2 (90.0)	71.4 (91.7)	71.5 (92.3)	71.7 (92.1)	71.9 (92.9)	72.6 (93.9)	70.3 (92.4)	69.6 (92.7)	73.9 (98.5)
Median (IQR)	27.0 (7.0–98.0)	30.0 (7.0–104.0)	31.0 (8.0–104.0)	30.0 (8.0–104.0)	31.0 (8.0–104.0)	31.0 (8.0–105.0)	29.0 (7.0–102.0)	27.0 (7.0–98.0)	29.0 (7.0–108.0)
**Gender**									
Female No. (%)	38009 (52.9%)	40350 (54.0%)	29842 (54.3%)	21115 (54.2%)	12639 (53.8%)	7405 (52.8%)	3407 (50.2%)	1720 (49.4%)	494 (46.6%)
Male No. (%)	60597 (47.1%)	34327 (46.0%)	25106 (45.7%)	17840 (45.8%)	10858 (46.2%)	6615 (47.2%)	3376 (49.8%)	1764 (50.6%)	565 (53.4%)

**HFRS:** Hospital frailty risk score; **NEWS2:** aggregate National Early Warning Score2; **LDT-EWS:** aggregate Laboratory Decision Tree Early Warning Score; **CCI:** Charlson Comorbidity Index; **CRP:** c-reactive protein test.

**Fig 2:**
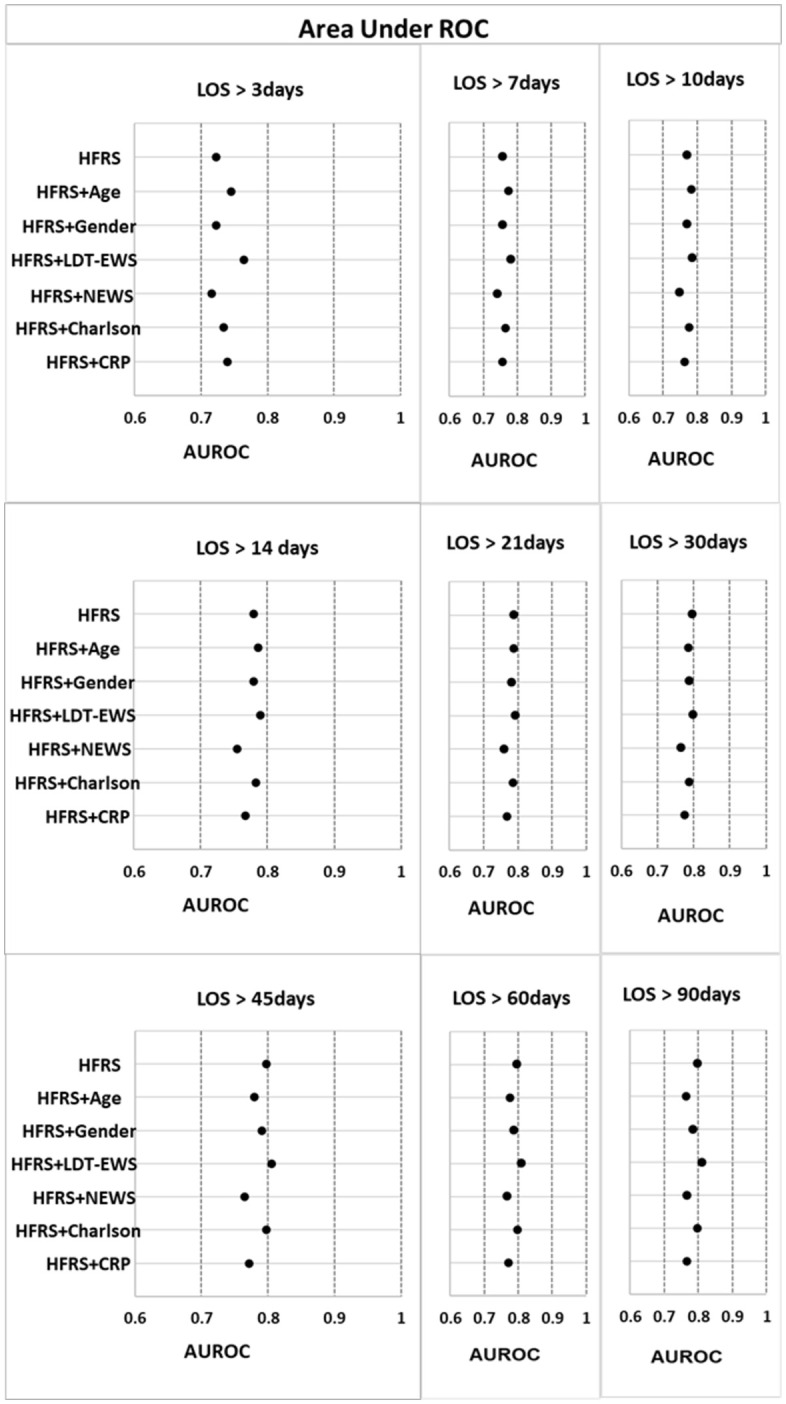
Area under ROC for 9 periods of prediction long length of stay.

Evaluation of the predictive power of each variable alone and HFRS combined with other variables is summarized in S3 Table and Fig 2. In general, the predictive power of crude HFRS (HFRS alone) increased with length of stay (AUROC = 0.723 for LOS > 3 days, rising to 0.798 for LOS > 90 days). Combining HFRS with age or CCI improved predictive ability of HFRS for LOS in excess of 3, 7, 10 and 14-days. However, in each LOS period, the AUROC value of the HFRS combined with LDT-EWS model was superior to each variable alone or models where HFRS was combined with any other variable. Combining HFRS with LDT-EWS gave AUROCs ranging from 0.764 to 0.810 (with all AUROCs being statistically significant, P < 0.001) showing that LDT-EWS significantly improves the predictive power of HFRS across all LOS periods.

To confirm the effectiveness of LDT-EWS, we investigated whether there were specific individual laboratory tests that influenced the result. However, the results showed that no single laboratory tests influenced HFRS as effectively as the LDT-EWS aggregate score. The results are detailed in S4 Table.

#### Validation results for LOS.

Applying our models to the validation dataset, we confirmed that HFRS + LDT-EWS models delivered the best results in terms of AUROC for all nine prediction periods.

Table 3 shows that for all eight validation samples according to admission year (2012–2019) there was fair or good discrimination. Also, for all four samples data according to age groups, discrimination was poor, fair, good or excellent. The two subsets according to gender had fair and good discrimination. Full details of the results are reported in S5-S7 Tables.

**Table 3:** Results of AUROC from Combining HFRS with LDT-EWS model for validation data.

Samples data	Range of AUROC
**Samples of data according to admission year**	
Sample data 2012 (n = 46642)	0.756–0.782
Sample data 2013 (n = 45922)	0.761–0.826
Sample data 2014 (n = 45668)	0.758–0.803
Sample data 2015 (n = 45745)	0.779–0.811
Sample data 2016 (n = 45973)	0.765–0.810
Sample data 2017 (n = 47431)	0.771–0.858
Sample data 2018 (n = 50375)	0.762–0.801
Sample data 2019 (n = 51160)	0.710–0.817
**Samples of data according to age**	
Age groups <45 years (n = 96963)	0.741–0.902
Age groups 45–64 years (n = 83952)	0.731–0.843
Age groups 65–84 years (n = 131042)	0.693–0.751
Age groups ≥85 years (n = 66959)	0.636–0.653
**Samples of data according to gender**	
Male data (n = 171008)	0.778–0.809
Female data (n = 207908)	0.745–0.782

Additional analyses were performed for multivariate models in S8 Table. The multivariate models offered lower results in terms of AUROC (ranging from 0.636 to 0.713) than the HFRS alone models. These results show that combining all variables in our study with the HFRS does not improve the predictive power of HFRS to predict a long length of stay.

Further analyses were performed on the dataset, after including patients who died in the hospital (S9 Table). The models offered results that were very similar to those above. These results demonstrate that HFRS+LDT-EWS significantly improves models’ performance to predict length of stay regardless of whether they died in hospital or not.

### HFRS and in-hospital mortality prediction

Patients’ characteristics according to in-hospital mortality periods are presented in Table 4. The mean HFRS was above 10, and most patients were categorised as intermediate or high risk of frailty, in all in-hospital mortality periods. The mean and median aggregate LDT-EWS was 4 and above. The mean and median aggregate NEWS were medium risk in all periods of in-hospital mortality. The mean CCI was about 11 and median was about 8. The mean CRP level was “elevated” and median was “moderately elevated” in all periods of in-hospital mortality.

**Table 4:** Characteristics of patients by predicted in-hospital mortality periods (n = 378,916).

	Prediction in-hospital mortality							
	3 days- mortality (n = 5,315)	7 days- mortality (n = 8,542)	10 days-mortality (n = 10,175)	14 days-mortality (n = 11,770)	30 days-mortality (n = 14,847)	60 days-mortality (n = 16,123)	90 days-mortality (n = 16,376)	6 month-mortality (n = 16,460)
**HFRS score**								
Mean (SD)	10.7 (9.9)	11.3 (10.1)	11.5 (10.2)	11.8 (10.3)	12.5 (10.5)	12.9 (10.6)	12.9 (10.7)	13.0 (10.7)
Median (IQR)	7.9 (3.3–15.4)	8.5 (3.8–16.1)	8.8 (4.0–16.3)	9.1 (4.2–16.7)	9.8 (4.6–17.7)	10.2 (4.8–18.2)	10.2 ((4.9–18.3)	10.3 (4.9–18.4)
**HFRS Category**								
Low	1834 (34.5)	2680 (31.4)	3063 (30.1)	3419 (29.0)	3949 (26.6)	4085 (25.3)	4119 (25.0)	4119 (25.0)
Intermediate	2089 (39.3)	3475 (40.7)	4189 (41.2)	4858 (41.3)	6098 (41.1)	6602 (41.0)	6735 (40.9)	6738 (40.9)
High	1392 (26.2)	2387 (27.9)	2923 (28.7)	3493 (29.7)	4800 (32.3)	5553 (33.9)	5606 (34.1)	5607 (34.1)
**Age in years**								
Mean (SD)	79.0 (13.0)	79.2 (12.7)	79.3 (12.5)	79.4 (12.5)	79.7 (12.3)	79.8 (12.2)	79.8 (12.2)	79.8 (12.2)
Median (IQR)	82.0 (72.0–89.0)	82.0 (72.0–88.0)	82.0 (72.0–88.0)	82.0 (72.0–88.0)	82.0 (73.0–89.0)	82.0 (73.0–89.0)	82.0 (73.0–89.0)	82.0 (73.0–89.0)
**LDT-EWS**								
Mean (SD)	4.2 (2.1)	4.2 (2.7)	4.2 (2.7)	4.2 (2.7)	4.2 (2.7)	4.2 (2.7)	4.2 (2.6)	4.2 (2.6)
Median (IQR)	4.0 (2.0–6.0)	4.0 (2.0–6.0)	4.0 (2.0–6.0)	4.0 (2.0–6.0)	4.0 (2.0–6.0)	4.0 (2.0–6.0)	4.0 (2.0–6.0)	4.0 (2.0–6.0)
**NEWS**								
Mean (SD)	5.4 (3.4)	4.9 (3.2)	4.7 (3.2)	4.5 (3.1)	4.2 (3.1)	4.1 (3.0)	4.1 (3.0)	4.1 (3.0)
Median (IQR)	5.0 (3.0–8.0)	5.0 (2.0–7.0)	4.0 (2.0–7.0)	4.0 (2.0–7.0)	4.0 (2.0–6.0)	4.0 (2.0–6.0)	4.0 (2.0–6.0)	4.0 (2.0–6.0)
**CCI**								
Mean (SD)	11.0 (13.3)	11.4 (13.6)	11.5 (13.7)	11.7 (13.9)	11.8 (13.9)	11.8 (13.9)	11.7 (13.9)	11.7 (13.9)
Median (IQR)	6.0 (0.0–18.0)	7.0 (0.0–18.0)	8.0 (0.0–19.0)	8.0 (0.0–19.0)	8.0 (0.0–20.0)	8.0 (0.0–20.0)	8.0 (0.0–20.0)	8.0 (0.0–20.0)
**CRP**								
Mean (SD)	111.7 (117.0)	110.1 (113.0)	108.4 (111.2)	107.1 (109.7)	103.5 (106.6)	101.4 (105.4)	101.0 (105.1)	100.9 (105.1)
Median (IQR)	72.0 (18–170.0)	75.0 (19–166.0)	75.0 (20–162.0)	73.0 (20–159.0)	71.0 (19–153.0)	68.0 (19–150.0)	68.0 (19–149.0)	68.0 (18–149·0)
**Gender**								
Female No· (%)	2662 (50.1%)	4236 (49.6%)	5005 (49.2%)	5758 (48.9%)	7183 (48.4%)	7810 (48.4%)	7925 (48.4%)	7963 (48.4%)
Male No· (%)	2653 (49.9%)	4306 (50.4%)	5170 (50.8%)	6012 (51.1%)	7664 (51.6%)	8313 (51.6%)	8451 (51.6%)	8497 (51.6%)

**HFRS:** Hospital frailty risk score; **Low** frailty risk is score <5; **Intermediate** frailty risk is score 5–15; **High** frailty risk is score >15; **LDT-EWS:** aggregate Laboratory Decision Tree Early Warning Score; **NEWS2:** aggregate National Early Warning Score2; **CCI:** Charlson Comorbidity Index; **CRP:** c-reactive protein test.

**Fig 3:**
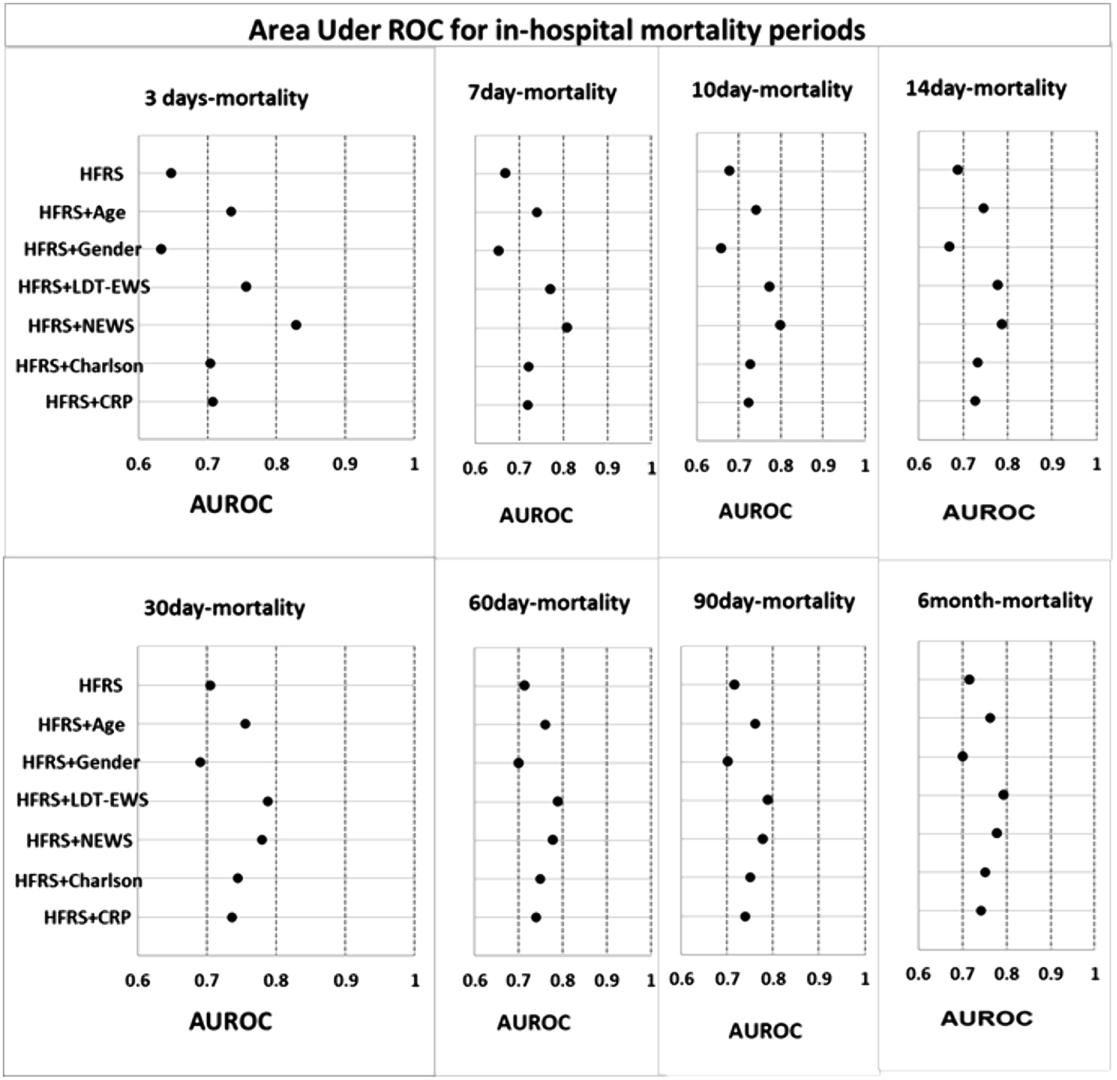
AUROC for 8 periods of prediction in-hospital mortality.

Evaluation of the predictive power of each variable alone and HFRS combined with one other variable is summarized in S10 Table and Fig 3. Although combining HFRS with age or LDT-EWS or CCI or CRP improves the predictive power of HFRS for predicting 3, 7, 10 and 14-day in-hospital mortality, the performance of HFRS combined with NEWS is superior to models where HFRS was combined with any other variable or any variable alone. For all periods longer than 14 days in-hospital mortality, combining HFRS with age or NEWS or CCI or CRP improves the ability of HFRS, but the performance of HFRS combined with LDT-EWS is superior to models where HFRS was combined with any other variable. All AUROCs for all periods of in-hospital mortality were statistically significant (P < 0.001). These results show that NEWS and LDT-EWS improve the predictive power of HFRS in predicting in-hospital mortality across all adult ages.

#### Validation results for in-hospital mortality.

Applying our models to the validation dataset, we confirmed that HFRS + NEWS models (for 3, 7, 10 and 14-day mortality) and HFRS + LDT-EWS models (for more than 14-day mortality) delivered the best results in terms of AUROC for samples according to admission year, age groups and gender.

Table 5 shows that for all eight validation samples according to admission year (2012–2019) there was fair and good discrimination. The four subsets according to age and the two subsets according to gender also had fair and good discrimination. Full details of the results are reported in S11-S13 Tables.

**Table 5:** Results of AUROC from Combining HFRS with NEWS or HFRS with LDT-EWS models for validation data.

Samples data	Range of AUROC for HFRS+NEWS for 3, 7, 10 and 14-day mortality	Range of AUROC for HFRS+LDT-EWS for more than 14 days mortality
**Samples of data according to admission year**		
Sample data 2012 (n = 46642)	0.802–0.837	0.803–0.805
Sample data 2013 (n = 45922)	0.798–0.848	0.800–0.806
Sample data 2014 (n = 45668)	0.793–0.847	0.793–0.800
Sample data 2015 (n = 45745)	0.789–0.806	0.797–0.799
Sample data 2016 (n = 45973)	0.781–0.845	0.790–0.796
Sample data 2017 (n = 47431)	0.779–0.832	0.790–0.801
Sample data 2018 (n = 50375)	0.790–0.823	0.784–0.786
Sample data 2019 (n = 51160)	0.786–0.800	0.798–0.801
**Samples of data according to age**		
Age groups <45 years (n = 96963)	0.888–0.899	0.877–0.895
Age groups 45–64 years (n = 83952)	0.821–0.854	0.824–0.828
Age groups 65–84 years (n = 131042)	0.749–0.797	0.735–0.738
Age groups ≥85 years (n = 66959)	0.724–0.786	0.700–0.710
**Samples of data according to gender**		
Male data (n = 171008)	0.791–0.833	0.782–0.784
Female data (n = 207908)	0.786–0.825	0.795–0.797

Additional analyses were performed for multivariate models (S14 Table). The multivariate models offered results that were close to those of simpler (i.e., HFRS+LDT-EWS) models. These results show that HFRS+NEWS significantly improves models’ performance to predict short term in-hospital mortality (up to 14 days) and HFRS+LDT-EWS significantly improves models’ performance to predict longer term in-hospital mortality.

## Discussion

Our study, involving hundreds of thousands of patients aged 16 years and above admitted to urgent care at a large secondary care hospital in the UK, found a significant association between HFRS and important clinical outcomes of LOS and in-hospital mortality. We also found that a laboratory test score (LDT-EWS) established shortly after admission combined with frailty risk score (HFRS) improved the prediction of LOS and in-patient mortality after 14 days more than other additional variables. Earlier mortality (less than 14 days) in hospital predictive power was most improved by the first physiological score (NEWS) at admission.

The HFRS has previously been associated with adverse outcomes for older people [13,14,16,19,21,39] including longer length of hospital stay. Our findings are similar for LOS in a broader group of non-electively admitted adults of all ages with the crude HFRS (HFRS alone) model having fair discrimination to predict LOS (between 0.723 to 0.798); as shown in Fig 2 and S3 Table. The predictive power of the HFRS for length of stay might partially be attributed to it including some significant healthcare events during hospitalisation (for example nosocomial infection).

Although identifying those at high risk of long LOS may be helpful to enable earlier interventions to reduce the risks associated with longer hospital stays such as deconditioning, hospital acquired infections and increased healthcare resource utilisation [3,40,41], the utility of the HFRS as an identification tool in clinical practice is limited by the inclusion of coding for the current admission, which is typically not available until after discharge. Previous studies have demonstrated limitations to utility in individuals [10].

The finding that AUROCs were improved for LOS of >3 days by age and CCI is in keeping with results in the original HFRS study that showed that discrimination of the model improved slightly by including patients’ characteristics [11]. Others have found HFRS to predict a prolonged LOS and that predictive value remains after adjustment for common variables such as age, sex, and the CCI or other comorbidity indices [11–15]. We found that age and CCI increased the predictive power of the HFRS, although still only with fair discrimination. Most importantly combining the aggregate LDT-EWS with the HFRS was consistently a more powerful predictor of length of stay than any other variables explored for all periods of length of stay, increasing the discrimination compared to HFRS alone, although this remained fair until LOS > 45 days when the performance increased to good.

Common laboratory test results can predict a higher risk of complication in hospital and community settings [26,31,32]. We found that separate specific laboratory tests did not influence the predictive power of HFRS, in contrast to laboratory tests together which were represented by LDT-EWS as one variable (S4 Table). There have been few studies of the HFRS, or other frailty risk indices, combined with laboratory tests for hospital inpatients. One study, Redfern et al, used the combination of LDT-EWS, NEWS and HFRS, along with the patient’s age, gender, and CCI in a multivariate model for the study outcomes of unplanned admission to the ICU and in-hospital mortality but LOS was not an outcome measured [42]. We looked at the potential for multivariate models to be more effective in predicting LOS but found these delivered lower results in terms of AUROC when compared to HFRS alone models or those combing HFRS with LDT-EWS (S8 Table). In another study, Sharma et al found that the model to predict hospital LOS remained significant after adding covariates (age, gender, CCI, Haemoglobin, and Creatinine) to the HFRS model but did not improve it [12].

HFRS alone has been found to be better than a warning score which incorporates the urea blood test result, at predicting LOS although it was not superior for mortality prediction [41]. So, whilst it is unlikely that individual blood test results improve the identification of those with frailty at risk of poor outcomes it may be that the larger number of blood tests that make up the LDT-EWS better reflect the spectrum and severity of illness that is represented in an undifferentiated cohort of hospital inpatients, including infection, that are not captured by one or two blood tests. The cumulative effect of abnormal laboratory tests with values that individually may not be predictive of outcomes, such as LOS or mortality, but when combined do so is recognised in a variety of settings, particularly in older people [43].

In this study we found that the HFRS combined with LDT-EWS remained a more effective predictor of length of stay than any other models whether those who died were included or not (S9 Table).

The majority of patients who died in-hospital, across all eight time periods, were categorized as having a high or intermediate risk of frailty. This is in keeping with other studies that found HFRS was an independent and good predictor of in-hospital mortality [39,44,45]. Our study extends this finding to a much broader group of patients – non-electively admitted adult patients of all ages although we found that HFRS had poor discrimination for predicting early mortality within 3 days when used alone.

Although HFRS has been widely used and validated in many countries for older patients [12,14,19,21,39,44,45], few studies have combined HFRS with other variables – common variables such as age, sex, and the CCI or other comorbidity indices – to assess improvement in its abilities to predict poor outcomes. A study found that HFRS with CCI offered only slightly better predictive power for 30-day in-hospital mortality for older people [46]. We have confirmed this finding in adults of all ages where combining HFRS with CCI did not improve (or only marginally improved) inpatient mortality prediction.

Early mortality prediction using HFRS alone was poor possibly because it does not capture illness severity or physiological parameters which might reflect the intensity of the acute stressor. Vital signs (NEWS) [23], LDT-EWS [22] and CRP [47] along with frailty measured using HFRS [11] are all associated with in-hospital mortality. We found that HFRS combined with NEWS was superior to other models, with good predictive ability for 3- and 7-day mortality and better, but still fair, for 10- and 14-day mortality (S10 Table and Fig 3). Küçükceran et al. and Alshibani et al. found that the NEWS2 was a significant predictor of in-hospital mortality [13,48] in older people who were admitted from Emergency Departments. A systematic review reported that NEWS2 has good predictive power in predicting early mortality in patients, but it has poor performance in predicting long-term mortality [49]. The additive information from combining a frailty index or Clinical Frailty Scale with an illness acuity score has previously been described in a study of older (average of about 80 years) general internal medicine referred patients where 30-day mortality likelihood was much higher in those with severe frailty who had higher acuity scores [50]. Others have found that a single admission physiological scoring system (NEWS2) was not helpful in predicting mortality in a group of older people with frailty and Covid 19, perhaps because it does not capture some of the risks associated with mortality such as delirium [51]. The HFRS does include delirium and common presenting complaints such as falls which are more common in those with frailty. LOS is complex, and whilst the HFRS is broad in component codes, it does not reflect all the factors that may affect LOS, including patient characteristics, in-hospital processes and the availability of non-hospital services such as social care.

For longer-term mortality, we found that models that combined HFRS with LDT-EWS were superior to other models, with fair discrimination for more than 14-day mortality across all adult ages (S10 Table and Fig 3). Others have reported that LDT-EWS was important in predicting mortality with good discrimination across all ages [22,43]. Adults with low early NEWS scores do subsequently die during a hospital admission and how to predict who is at higher risk of this is not clear [52]. Indices that combine laboratory tests and frailty can indicate a higher risk of mortality in a variety of settings and patient cohorts [43,53,54]. Whilst the LDT-EWS includes far fewer laboratory tests than typical frailty indices it may be that in an acute setting it contributes enough cumulative information to risk of mortality than individual laboratory tests.

This study has several strengths. It is the ﬁrst study reporting factors that influence the power of HFRS to predict LOS and in-hospital mortality across all adult ages in an unselected non-elective cohort. The study used an optimal construction of the HFRS (from index admission through previous admissions over 2 years) [14]. Previous studies have used a cut-off of 10 or more days to indicate prolonged LOS and 30-day in-hospital mortality, which may not be clinically relevant enough to capture very long hospital stays or in-hospital mortality associated with patient complexity. We have used nine different periods of time for LOS and eight different periods for in-hospital mortality. We used a laboratory warning score (comprising a small number of laboratory tests) and vital signs typically available for patients admitted overnight. We also utilised multiple sampling methods and validation checks of the results.

While this study demonstrated that a higher HFRS is associated with an increasing risk of longer LOS and in-hospital mortality across all adult ages, it does have some limitations. This study uses a large amount of data, but it is restricted to one hospital, so further research is warranted to see whether the results are replicated in different settings. Also, death was based on the patient’s clinical status at the time of discharge; we could not link records to the General Register Office (death records) to know patients who died (perhaps, shortly) after discharge. We selected a broad non-elective presentation cohort of patients. This most likely reflects the situation of patients living with frailty who often present acutely to hospital with a number of simultaneous complaints and have multiple diagnoses. Future work could focus on whether there is enhanced predictive ability in specific cohorts, for example those with a hip fracture.

HFRS was calculated using the original methodology which includes index admission coding, which would not be known at the point of admission, limiting clinical applicability of the HFRS in an acute admission. Previous studies have demonstrated limitations to utility in individuals [10]. A modified HFRS (by excluding index admission from HFRS calculation) has been reported to be correlated with the HFRS and the Clinical Frailty Scale in older patients and had similar associations with outcomes of longer length of stay and mortality [55]. We did not undertake similar analysis to know whether similar findings would apply in our cohort of all ages patients. The calculation of HFRS and the other indices used is likely to be technically easy to apply in hospital systems, however this is reliant on electronic coding of diagnoses, observations and blood test results.

## Conclusions

We conclude that the LDT-EWS can significantly improve the predictive power of HFRS to predict LOS in the hospital whilst the NEWS and LDT-EWS can improve the predictive power of HFRS to predict in-hospital mortality. HFRS combined with NEWS was a good predictor in short-term in-hospital mortality across all adult ages, and HFRS with LDT-EWS for longer-term mortality in all adults admitted urgently to hospital. Therefore, the applications of these results are particularly useful for retrospectively characterising at-risk hospital populations on a macro scale or for use in epidemiological research. Further research is needed and could investigate a number of areas including: whether these findings are replicated in other hospitals, if the findings are replicated in different groups of patients (such as those admitted electively), the performance of an HFRS based only on previous admissions (by excluding current admission), whether targeted intervention in those at higher risk of longer LOS or inpatient mortality using the HFRS+NEWS and HFRS+LDT-EWS results in improved outcomes, and the utility of the combined score in a clinical setting to support identification and guide service development approaches.

## Supporting information

S1 Table Cross validation. (DOCX)

S2 Table Speciality of discharge. (DOCX)

S3 Table Results of AUROC for 9 period of longer length of stay for each variable alone and HFRS combined with one other variable. (DOCX)

S4 Table Results of AUROC for HFRS with separate laboratory tests models, and HFRS with LDT-EWS models for 9 periods of LOS. (DOCX)

S5 Table S5a-S5h Tables. Results of AUROC for 9 period of LOS according to admission year.
(DOCX)

S6 Table S6a-S6d Tables. Results of AUROC for 9 period of LOS according to age groups.
(DOCX)

S7 Table S7a-S7b Tables. Results of AUROC for 9 period of LOS according to gender.
(DOCX)

S8 Table Results of AUROC for 9 periods of longer length of stay for multivariate models. (DOCX)

S9 Table Results of AUROC for 9 periods of length of stay for data after included patients who died in hospital for each variable alone and HFRS combined with one other variable. (DOCX)

S10 Table AUROC for 8 prediction in-hospital mortality for each variable alone and HFRS combined with one other variable. (DOCX)

S11 Table AUROC for HFRS combined with one other variable according to admission year for 8 periods of in-hospital mortality. (DOCX)

S12 Table AUROC for HFRS alone and HFRS combined with one other variable according to age groups for 8 periods of in-hospital mortality. (DOCX)

S13 Table AUROC for HFRS alone and HFRS combined with one other variable according to gender for 8 periods of in-hospital mortality. (DOCX)

S14 Table AUROC for multivariate models for 8 prediction in-hospital mortality. (DOCX)

## References

[1] Morley JE, Vellas B, van Kan GA, Anker SD, Bauer JM, Bernabei R, et al. Frailty consensus: a call to action. J Am Med Dir Assoc. 2013;14(6):392–7. doi: 10.1016/j.jamda.2013.03.022 23764209 PMC4084863

[2] Nguyen TN, Cumming RG, Hilmer SN. The impact of frailty on mortality, length of stay and re-hospitalisation in older patients with atrial fibrillation. Heart Lung Circ. 2016;25(6):551–7. doi: 10.1016/j.hlc.2015.12.002 26809464

[3] Han L, Clegg A, Doran T, Fraser L. The impact of frailty on healthcare resource use: a longitudinal analysis using the Clinical Practice Research Datalink in England. Age Ageing. 2019;48(5):665–71. doi: 10.1093/ageing/afz088 31297511

[4] Richards SJG, D’Souza J, Pascoe R, Falloon M, Frizelle FA. Prevalence of frailty in a tertiary hospital: a point prevalence observational study. PLoS One. 2019;14(7):e0219083. doi: 10.1371/journal.pone.0219083 31260483 PMC6602419

[5] Knight T, Atkin C, Martin FC, Subbe C, Holland M, Cooksley T, et al. Frailty assessment and acute frailty service provision in the UK: results of a national “day of care” survey. BMC Geriatr. 2022;22(1):19. doi: 10.1186/s12877-021-02679-9 34979956 PMC8721940

[6] Kehler DS, Ferguson T, Stammers AN, Bohm C, Arora RC, Duhamel TA, et al. Prevalence of frailty in Canadians 18-79 years old in the Canadian Health Measures Survey. BMC Geriatr. 2017;17(1):28. doi: 10.1186/s12877-017-0423-6 28107813 PMC5251297

[7] Loecker C, Schmaderer M, Zimmerman L. Frailty in young and middle-aged adults: an integrative review. J Frailty Aging. 2021;10(4):327–33. doi: 10.14283/jfa.2021.14 34549246

[8] Gordon EH, Peel NM, Hubbard RE, Reid N. Frailty in younger adults in hospital. QJM. 2023;116(10):845–9. doi: 10.1093/qjmed/hcad173 37467071 PMC10593383

[9] Rockwood K, Song X, Mitnitski A. Changes in relative fitness and frailty across the adult lifespan: evidence from the Canadian National Population Health Survey. CMAJ. 2011;183(8):E487–94. doi: 10.1503/cmaj.101271 21540166 PMC3091935

[10] De Geer L, Fredrikson M, Chew MS. Frailty is a stronger predictor of death in younger intensive care patients than in older patients: a prospective observational study. Ann Intensive Care. 2022;12(1):120. doi: 10.1186/s13613-022-01098-2 36586004 PMC9803889

[11] Gilbert T, Neuburger J, Kraindler J, Keeble E, Smith P, Ariti C, et al. Development and validation of a Hospital Frailty Risk Score focusing on older people in acute care settings using electronic hospital records: an observational study. Lancet. 2018;391(10132):1775–82. doi: 10.1016/S0140-6736(18)30668-8 29706364 PMC5946808

[12] Sharma Y, Horwood C, Hakendorf P, Shahi R, Thompson C. External validation of the Hospital Frailty-Risk Score in predicting clinical outcomes in older heart-failure patients in Australia. J Clin Med. 2022;11(8):2193. doi: 10.3390/jcm11082193 35456288 PMC9028959

[13] Alshibani A, Coats T, Maynou L, Lecky F, Banerjee J, Conroy S. A comparison between the clinical frailty scale and the Hospital Frailty Risk Score to risk stratify older people with emergency care needs. BMC Emerg Med. 2022;22(1):171. doi: 10.1186/s12873-022-00730-5 36284266 PMC9598033

[14] Street A, Maynou L, Gilbert T, Stone T, Mason S, Conroy S. The use of linked routine data to optimise calculation of the Hospital Frailty Risk Score on the basis of previous hospital admissions: a retrospective observational cohort study. Lancet Healthy Longev. 2021;2(3):e154–62. doi: 10.1016/S2666-7568(21)00004-0 33733245 PMC7934406

[15] Gouda P, Wang X, Youngson E, McGillion M, Mamas MA, Graham MM. Beyond the revised cardiac risk index: validation of the Hospital Frailty Risk Score in non-cardiac surgery. PLoS One. 2022;17(1):e0262322. doi: 10.1371/journal.pone.0262322 35045122 PMC8769314

[16] Kundi H, Noseworthy PA, Valsdottir LR, Shen C, Yao X, Yeh RW, et al. Relation of frailty to outcomes after catheter ablation of atrial fibrillation. Am J Cardiol. 2020;125(9):1317–23. doi: 10.1016/j.amjcard.2020.01.049 32147090

[17] Kutrani H, Briggs J, Prytherch D, Spice C. Using the Hospital Frailty Risk Score to predict length of stay across all adult ages. PLoS One. 2025;20(1):e0317234. doi: 10.1371/journal.pone.0317234 39847554 PMC11756769

[18] Kutrani H, Briggs J, Prytherch D, Andrikopoulou E, Spice C. Using the hospital frailty risk score to predict in-hospital mortality across all ages. Intelligent health systems – from technology to data and knowledge. ISO Press; 2025. pp. 562–6. doi: 10.3233/SHTI250400 40380510

[19] Eckart A, Hauser SI, Haubitz S, Struja T, Kutz A, Koch D, et al. Validation of the hospital frailty risk score in a tertiary care hospital in Switzerland: results of a prospective, observational study. BMJ Open. 2019;9(1):e026923. doi: 10.1136/bmjopen-2018-026923 30647051 PMC6340447

[20] Soong J, Bell D, Poots AJ. The challenges of using the Hospital Frailty Risk Score. The Lancet. 2018;:2692. doi: 10.1016/S0140-6736(18)32426-7 30587361

[21] Gilbert T, Cordier Q, Polazzi S, Bonnefoy M, Keeble E, Street A, et al. External validation of the Hospital Frailty Risk Score in France. Age Ageing. 2022;51(1):afab126. doi: 10.1093/ageing/afab126 34185827 PMC8753041

[22] Jarvis SW, Kovacs C, Badriyah T, Briggs J, Mohammed MA, Meredith P, et al. Development and validation of a decision tree early warning score based on routine laboratory test results for the discrimination of hospital mortality in emergency medical admissions. Resuscitation. 2013;84(11):1494–9. doi: 10.1016/j.resuscitation.2013.05.018 23732049

[23] Royal College of Physicians. National Early Warning Score (NEWS) 2: Standardising the assessment of acute-illness severity in the NHS. London: RCP; 2017. www.rcplondon.ac.uk

[24] Elliott M, Endacott R. The clinical neglect of vital signs’ assessment: an emerging patient safety issue? Contemp Nurse. 2022;58(4):249–52. doi: 10.1080/10376178.2022.2109494 35924342

[25] Prytherch DR, Briggs JS, Weaver PC, Schmidt P, Smith GB. Measuring clinical performance using routinely collected clinical data. Med Inform Internet Med. 2005;30(2):151–6. doi: 10.1080/14639230500298966 16338803

[26] Huang S, Wang Y, Chen L, Chen X. Use of a frailty index based upon routine laboratory data to predict complication and mortality in older community-acquired pneumonia patients. Arch Gerontol Geriatr. 2022;101:104692. doi: 10.1016/j.archger.2022.104692 35349877

[27] Kim SH, Choi HS, Jin ES, Choi H, Lee H, Lee S-H, et al. Predicting severe outcomes using national early warning score (NEWS) in patients identified by a rapid response system: a retrospective cohort study. Sci Rep. 2021;11(1):18021. doi: 10.1038/s41598-021-97121-w 34504146 PMC8429773

[28] Pugazhvannan CR, Vanidassane I, Pownraj D, Kandasamy R, Basheer A. National Early Warning Score 2 (NEWS2) to predict poor outcome in hospitalised COVID-19 patients in India. PLoS One. 2021. doi: 10.1371/journal.pone.0261376 PMC8673675 34910789

[29] Prytherch DR, Sirl JS, Schmidt P, Featherstone PI, Weaver PC, Smith GB. The use of routine laboratory data to predict in-hospital death in medical admissions. Resuscitation. 2005;66(2):203–7. doi: 10.1016/j.resuscitation.2005.02.011 15955609

[30] Yeong E-K, Tung K-Y, Chang C-H, Tsai S-J. The relationships between routine admission blood tests and burn size, and length of stay in intensive care unit. J Formos Med Assoc. 2022;121(12):2512–9. doi: 10.1016/j.jfma.2022.05.012 35701304

[31] Bopche R, Gustad LT, Afset JE, Ehrnström B, Damås JK, Nytrø Ø. In-hospital mortality, readmission, and prolonged length of stay risk prediction leveraging historical electronic patient records. JAMIA Open. 2024;7(3):ooae074. doi: 10.1093/jamiaopen/ooae074 39282081 PMC11401612

[32] Cournane S, Byrne D, O’Riordan D, Silke B. Factors associated with length of stay following an emergency medical admission. Eur J Intern Med. 2015;26(4):237–42. doi: 10.1016/j.ejim.2015.02.017 25743060

[33] Chung HS, Choi Y, Lim JY, Kim K, Choi YH, Lee DH, et al. The clinical frailty scale improves risk prediction in older emergency department patients: a comparison with qSOFA, NEWS2, and REMS. Sci Rep. 2025;15(1):12584. doi: 10.1038/s41598-025-97764-z 40221594 PMC11993572

[34] Kutrani H, Briggs J, Prytherch D, Spice C. Age does not improve the predictive ability of the Hospital Frailty Risk Score for length of stay. PLoS One. 2025;20(9):e0330930. doi: 10.1371/journal.pone.0330930 40924688 PMC12419641

[35] Yan Y-M, Gao J, Jin P-L, Lu J-J, Yu Z-H, Hu Y. C-reactive protein as a non-linear predictor of prolonged length of intensive care unit stay after gastrointestinal cancer surgery. World J Clin Cases. 2022;10(31):11381–90. doi: 10.12998/wjcc.v10.i31.11381 36387784 PMC9649545

[36] Carteron N. C-Reactive Protein Level Chart for Rheumatoid Arthritis. 2023. pp. 10. [cited 2023 Nov 2]. Available from: https://www.healthline.com/health/rheumatoid-arthritis-crp-levels

[37] Clark DE, Ryan LM. Concurrent prediction of hospital mortality and length of stay from risk factors on admission. Health Serv Res. 2002;37(3):631–45. doi: 10.1111/1475-6773.00041 12132598 PMC1434655

[38] Nahm FS. Receiver operating characteristic curve: overview and practical use for clinicians. Korean J Anesthesiol. 2022;75(1):25–36. doi: 10.4097/kja.21209 35124947 PMC8831439

[39] Ramai D, Dang-Ho KP, Kewalramani A, Bandaru P, Sacco R, Giacomelli L. Hospital Frailty Risk Score is independently associated with mortality and encephalopathy. Biomedicines. 2021:1693. doi: 10.3390/biomedicines 34829921 PMC8615905

[40] Baek H, Cho M, Kim S, Hwang H, Song M, Yoo S. Analysis of length of hospital stay using electronic health records: a statistical and data mining approach. PLoS One. 2018;13(4):e0195901. doi: 10.1371/journal.pone.0195901 29652932 PMC5898738

[41] Rosario BH, Quah JL, Chang TY, Barrera VC, Lim A, Sim LE, et al. Validation of the Hospital Frailty Risk Score in older adults hospitalized with community-acquired pneumonia. Geriatr Gerontol Int. 2024;24 Suppl 1(Suppl 1):135–41. doi: 10.1111/ggi.14697 37846810 PMC11503533

[42] Redfern OC, Harford M, Gerry S, Prytherch D, Watkinson PJ. Frailty and unplanned admissions to the intensive care unit: a retrospective cohort study in the UK. Intensive Care Med. 2020;46(7):1512–3. doi: 10.1007/s00134-020-06020-7 32240348

[43] Sapp DG, Cormier BM, Rockwood K, Howlett SE, Heinze SS. The frailty index based on laboratory test data as a tool to investigate the impact of frailty on health outcomes: a systematic review and meta-analysis. Age Ageing. 2023;52(1):afac309. doi: 10.1093/ageing/afac309 36626319 PMC9831271

[44] Sohal A, Chaudhry H, Kohli I, Gupta G, Singla P, Sharma R, et al. Hospital frailty risk score predicts worse outcomes in patients with chronic pancreatitis. Ann Gastroenterol. 2023;36(1):73–80. doi: 10.20524/aog.2022.0765 36593805 PMC9756028

[45] Kilkenny MF, Phan HT, Lindley RI, Kim J, Lopez D, Dalli LL, et al. Utility of the Hospital Frailty Risk Score derived from administrative data and the association with stroke outcomes. Stroke. 2021;52(9):2874–81. doi: 10.1161/STROKEAHA.120.033648 34134509

[46] Gilbert T, Cordier Q, Polazzi S, Street A, Conroy S, Duclos A. Combining the Hospital Frailty Risk Score with the Charlson and Elixhauser multimorbidity indices to identify older patients at risk of poor outcomes in acute care. Med Care. 2024;62(2):117–24. doi: 10.1097/MLR.0000000000001962 38079225 PMC10773558

[47] Maluf CB, Barreto SM, Giatti L, Ribeiro AL, Vidigal PG, Azevedo DRM, et al. Association between C reactive protein and all-cause mortality in the ELSA-Brasil cohort. J Epidemiol Community Health. 2020;74(5):421–7. doi: 10.1136/jech-2019-213289 32102838 PMC7307658

[48] Küçükceran K, Ayrancı MK, Koçak S, Girişgin AS, Dündar ZD, Ataman S, et al. An evaluation of the National Early Warning Score 2 and the laboratory data decision tree early warning score in predicting mortality in geriatric patients. J Emerg Med. 2024;66(3):e284–92. doi: 10.1016/j.jemermed.2023.10.012 38278676

[49] Wei S, Xiong D, Wang J, Liang X, Wang J, Chen Y. The accuracy of the National Early Warning Score 2 in predicting early death in prehospital and emergency department settings: a systematic review and meta-analysis. Ann Transl Med. 2023;11(2):95. doi: 10.21037/atm-22-6587 36819553 PMC9929743

[50] Pulok MH, Theou O, van der Valk AM, Rockwood K. The role of illness acuity on the association between frailty and mortality in emergency department patients referred to internal medicine. Age Ageing. 2020;49(6):1071–9. doi: 10.1093/ageing/afaa089 32392289 PMC7583513

[51] Rønningen PS, Walle-Hansen MM, Ihle-Hansen H, Andersen EL, Tveit A, Myrstad M. Impact of frailty on the performance of the National Early Warning Score 2 to predict poor outcome in patients hospitalised due to COVID-19. BMC Geriatr. 2023;23(1):134. doi: 10.1186/s12877-023-03842-0 36890484 PMC9994778

[52] Holland M, Kellett J. A systematic review of the discrimination and absolute mortality predicted by the National Early Warning Scores according to different cut-off values and prediction windows. Eur J Intern Med. 2022;98:15–26. doi: 10.1016/j.ejim.2021.12.024 34980504

[53] Rockwood K, Mcmillan M, Mitnitski AB, Howlett SE, Lekan DA, Jacobs JM. A frailty index based on common laboratory tests in comparison with a clinical frailty index for older adults in long term care facilities. 2015;55:235. https://research.ebsco.com/linkprocessor/plink?id=83658a8d-42e8-3c9f-9e6f-1144ca9763fd 10.1016/j.jamda.2015.03.027 25952475

[54] Bai W, Hao B, Xu L, Qin J, Xu W, Qin L. Frailty index based on laboratory tests improves prediction of short-and long-term mortality in patients with critical acute myocardial infarction. Front Med (Lausanne). 2022;9:1070951. doi: 10.3389/fmed.2022.1070951 36561712 PMC9763273

[55] Sim L, Chang TY, Htin KK, Lim A, Selvaratnam T, Conroy S, et al. Modified Hospital Frailty Risk Score (mHFRS) as a tool to identify and predict outcomes for hospitalised older adults at risk of frailty. J Frailty Sarcopenia Falls. 2024;9(4):235–48. doi: 10.22540/JFSF-09-235 39635564 PMC11613971

