# Supplementary material for: Vital signs and common blood tests improve the predictive power of the Hospital Frailty Risk Score to predict poor outcomes across all adult ages
Source: PLoS One. 2026 May 5;21(5):e0348669. doi: 10.1371/journal.pone.0348669 (PMC13143055; doi:10.1371/journal.pone.0348669)
Supplement: S1 Table — (DOCX) [file pone.0348669.s001.docx]

**S1 Table. Cross validation**

Prediction models need to determine the quality of the models by using model validation and evaluate the stability of the models which refers to whether the model actually achieves the same results or not. Also, if different samples of training and testing datasets could have different results or not.

We generated many data for validation experiments which include the following:

- 8 samples according to admission year, we generated sample data for each year starting from 2012 to 2019.

- 4 samples according to age.

- 2 samples according to gender.

|  | **Sample data according to admission year** | Number of admissions |
| --- | --- | --- |
| 1 | patients who were admitted to QA hospital in 2012 (sample data 2012) | 46642 |
| 2 | patients who were admitted to QA hospital in 2013 (sample data 2013) | 45922 |
| 3 | patients who were admitted to QA hospital in 2014 (sample data 2014) | 45668 |
| 4 | patients who were admitted to QA hospital in 2015 (sample data 2015) | 45745 |
| 5 | patients who were admitted to QA hospital in 2016 (sample data 2016) | 45973 |
| 6 | patients who were admitted to QA hospital in 2017 (sample data 2017) | 47431 |
| 7 | patients who were admitted to QA hospital in 2018 (sample data 2018) | 50375 |
| 8 | patients who were admitted to QA hospital in 2018 (sample data 2019) | 51160 |
|  | **Sample data according to age** | Number of admissions |
| 1 | patients aged less than 45 years (<45 yrs data) | 96963 |
| 2 | patients aged from 45-64 years (45-64 yrs data) | 83952 |
| 3 | patients aged from 65-84 years (65-84 yrs data) | 131042 |
| 4 | patients aged from 85 and above (≥85 yrs data) | 66959 |
|  | **Sample data according to gender** | Number of admissions |
| 1 | males only | 171008 |
| 2 | females only | 207908 |
