# Supplementary material for: Vital signs and common blood tests improve the predictive power of the Hospital Frailty Risk Score to predict poor outcomes across all adult ages
Source: PLoS One. 2026 May 5;21(5):e0348669. doi: 10.1371/journal.pone.0348669 (PMC13143055; doi:10.1371/journal.pone.0348669)
Supplement: S2 Table — (DOCX) [file pone.0348669.s002.docx]

**S2 Table: Speciality of discharge**

| **Discharge speciality** | **Number** | **%** |
| --- | --- | --- |
| General medicine | 154090 | 40.67 |
| Accident & emergency | 46240 | 12.2 |
| Gynaecology | 25818 | 6.81 |
| Trauma & orthopaedic surgery | 18331 | 4.84 |
| Hips | 18201 | 4.8 |
| General surgery | 17641 | 4.66 |
| Cardiology | 13471 | 3.56 |
| Upper gastrointestinal surgery | 13347 | 3.52 |
| Nephrology | 11161 | 2.95 |
| Urology | 10059 | 2.65 |
| Stroke medicine | 9592 | 2.53 |
| Thoracic medicine | 6041 | 1.59 |
| Medical oncology | 5952 | 1.57 |
| Geriatric medicine | 3364 | 0.89 |
| Clinical oncology | 3158 | 0.83 |
| Haematology - clinical | 2710 | 0.72 |
| EM elderly acute | 1735 | 0.46 |
| Others | 18,008 | 4.75 |
| Total |  | 100 |
