# Supplementary material for: Vital signs and common blood tests improve the predictive power of the Hospital Frailty Risk Score to predict poor outcomes across all adult ages
Source: PLoS One. 2026 May 5;21(5):e0348669. doi: 10.1371/journal.pone.0348669 (PMC13143055; doi:10.1371/journal.pone.0348669)
Supplement: S3 Table — (DOCX) [file pone.0348669.s003.docx]

**S3 Table. Results of AUROC** **for 9 period of longer length of stay for each variable alone and HFRS combined with one other variable**

| **Outcomes** | **LOS>3-day** | **LOS>7-day** | **LOS>10-day** | **LOS>14-day** | **LOS>21-day** | **LOS>30-day** | **LOS>45-day** | **LOS>60-day** | **LOS>90-day** |
| --- | --- | --- | --- | --- | --- | --- | --- | --- | --- |
|  | AUROC  (95% CI) | AUROC  (95% CI) | AUROC  (95% CI) | AUROC  (95% CI) | AUROC  (95% CI) | AUROC  (95% CI) | AUROC  (95% CI) | AUROC  (95% CI) | AUROC  (95% CI) |
| **HFRS alone** | **0.723** | **0.757** | **0.770** | **0.779** | **0.789** | **0.796** | **0.798** | **0.797** | **0.798** |
|  | **(0.719-0.726)** | **(0.754-0.761)** | **(0.766-0.773)** | **(0.775-0.783)** | **(0.784-0.794)** | **(0.790-0.801)** | **(0.790-0.805)** | **(0.786-0.807)** | **(0.781-0.816)** |
| **Age alone** | 0.712 | 0.734 | 0.741 | 0.743 | 0.738 | 0.725 | 0.712 | 0.690 | 0.637 |
|  | (0.709-0.715) | (0.731-0.738) | (0.737-0.745) | (0.738-0.747) | (0.732-0.743) | (0.719-0.732) | (0.703-0.722) | (0.676-0.704) | (0.61-0.664) |
| **Gender alone** | 0.517 | 0.506 | 0.503 | 0.501 | 0.501 | 0.507 | 0.521 | 0.524 | 0.530 |
|  | (0.514-0.52) | (0.502-0.509) | (0.499-0.507) | (0.496-0.506) | (0.495-0.507) | (0.499-0.515) | (0.51-0.532) | (0.508-0.539) | (0.502-0.558) |
| **LDT-EWS alone** | 0.724 | 0.719 | 0.713 | 0.705 | 0.693 | 0.683 | 0.679 | 0.662 | 0.663 |
|  | (0.721-0.727) | (0.715-0.722) | (0.709-0.717) | (0.7-0.71) | (0.687-0.699) | (0.675-0.691) | (0.668-0.691) | (0.647-0.678) | (0.634-0.692) |
| **NEWS alone** | 0.615 | 0.607 | 0.603 | 0.599 | 0.591 | 0.590 | 0.573 | 0.573 | 0.588 |
|  | (0.611-0.619) | (0.603-0.611) | (0.598-0.608) | (0.594-0.605) | (0.584-0.598) | (0.581-0.599) | (0.56-0.586) | (0.555-0.591) | (0.556-0.621) |
| **CCI alone** | 0.615 | 0.619 | 0.619 | 0.614 | 0.606 | 0.584 | 0.569 | 0.539 | 0.513 |
|  | (0.612-0.618) | (0.615-0.623) | (0.614-0.623) | (0.609-0.619) | (0.599-0.612) | (0.576-0.593) | (0.556-0.581) | (0.522-0.555) | (0.484-0.542) |
| **CRP alone** | 0.666 | 0.647 | 0.637 | 0.628 | 0.620 | 0.619 | 0.608 | 0.608 | 0.612 |
|  | (0.662-0.671) | (0.642-0.652) | (0.631-0.643) | (0.621-0.635) | (0.612-0.629) | (0.608-0.63) | (0.592-0.624) | (0.585-0.631) | 0.798 |
| **HFRS+ age** | 0.745 | 0.774 | 0.782 | 0.786 | 0.787 | 0.784 | 0.780 | 0.775 | 0.764 |
|  | (0.742-0.748) | (0.770-0.777) | (0.779-0.786) | (0.782-0.79) | (0.782-0.792) | (0.778-0.79) | (0.772-0.789) | (0.763-0.787) | (0.742-0.786) |
| **HFRS+ gender** | 0.722 | 0.758 | 0.77 | 0.779 | 0.782 | 0.787 | 0.791 | 0.787 | 0.785 |
|  | (0.719-0.725) | (0.754-0.761) | (0.766-0.774) | (0.775-0.783) | (0.780-0.793) | (0.784-0.800) | (0.783-0.799) | (0.776-0.799) | (0.765-0.805) |
| **HFRS+ LDT-EWS** | **0.764** | **0.782** | **0.786** | **0.789** | **0.793** | **0.798** | **0.805** | **0.810** | **0.810** |
|  | **(0.762-0.767)** | **(0.778-0.785)** | **(0.783-0.79)** | **(0.786-0.793)** | **(0.788-0.797)** | **(0.787-0.799)** | **(0.786-0.808)** | **(0.779-0.815)** | **(0.773-0.809)** |
| **HFRS+ NEWS** | 0.716 | 0.741 | 0.749 | 0.755 | 0.760 | 0.766 | 0.765 | 0.767 | 0.767 |
|  | (0.713-0.72) | (0.737-0.744) | (0.745-0.753) | (0.751-0.76) | (0.755-0.766) | (0.760-0.773) | (0.757-0.774) | (0.755-0.778) | (0.747-0.787) |
| **HFRS+ CCI** | 0.734 | 0.765 | 0.776 | 0.783 | 0.785 | 0.786 | 0.798 | 0.797 | 0.796 |
|  | (0.731-0.737) | (0.762-0.769) | (0.772-0.779) | (0.779-0.787) | (0.783-0.796) | (0.782-0.801) | (0.790-0.806) | (0.786-0.807) | (0.779-0.817) |
| **HFRS+CRP** | 0.74 | 0.757 | 0.762 | 0.767 | 0.769 | 0.774 | 0.772 | 0.772 | 0.765 |
|  | (0.736-0.744) | (0.753-0.762) | (0.757-0.767) | (0.762-0.772) | (0.763-0.776) | (0.766-0.782) | (0.760-0.783) | (0.757-0.788) | (0.738-0.792) |

**HFRS:** Hospital frailty risk score; **NEWS:** aggregate National Early Warning Score; **LDT-EWS:** aggregate Laboratory Decision Tree Early Warning Score; **CCI:** Charlson Comorbidity Index; **CRP:** c-reactive protein test
