# Supplementary material for: Vital signs and common blood tests improve the predictive power of the Hospital Frailty Risk Score to predict poor outcomes across all adult ages
Source: PLoS One. 2026 May 5;21(5):e0348669. doi: 10.1371/journal.pone.0348669 (PMC13143055; doi:10.1371/journal.pone.0348669)
Supplement: S4 Table — (DOCX) [file pone.0348669.s004.docx]

**S4 Table. Results of AUROC** **for HFRS with separate laboratory tests models, and HFRS with LDT-EWS models for 9 periods of LOS**

| **Models** | **LOS>3 day** | **LOS>7 day** | **LOS>10 day** | **LOS>14 day** | **LOS>21 day** | **LOS>30day** | **LOS>45day** | **LOS>60day** | **LOS>90day** |
| --- | --- | --- | --- | --- | --- | --- | --- | --- | --- |
|  | AUROC | AUROC | AUROC | AUROC | AUROC | AUROC | AUROC | AUROC | AUROC |
| HFRS+ LDT-EWS | **0.764** | **0.782** | **0.786** | **0.789** | **0.793** | **0.798** | **0.805** | **0.810** | **0.810** |
| HFRS+ Na | 0.692 | 0.708 | 0.714 | 0.708 | 0.701 | 0.687 | 0.677 | 0.659 | 0.582 |
| HFRS+ ALB | 0.735 | 0.749 | 0.752 | 0.744 | 0.738 | 0.727 | 0.716 | 0.695 | 0.661 |
| HFRS+ K | 0.677 | 0.695 | 0.701 | 0.696 | 0.693 | 0.681 | 0.671 | 0.653 | 0.582 |
| HFRS+ CR | 0.689 | 0.707 | 0.712 | 0.706 | 0.702 | 0.69 | 0.682 | 0.665 | 0.586 |
| HFRS+ U | 0.702 | 0.72 | 0.724 | 0.718 | 0.715 | 0.701 | 0.694 | 0.678 | 0.626 |
| HFRS+ WCC | 0.699 | 0.711 | 0.715 | 0.711 | 0.707 | 0.694 | 0.678 | 0.65 | 0.614 |
| HFRS+GB | 0.673 | 0.688 | 0.712 | 0.710 | 0.708 | 0.697 | 0.678 | 0.654 | 0.636 |
| HFRS+ CRP | **0.740** | **0.757** | **0.762** | **0.767** | **0.769** | **0.774** | **0.772** | **0.772** | **0.765** |

**LDT-EWS:** Laboratory Decision Tree Early Warning Score; **Na:** Sodium; **ALB:** Albumin; **K:** Potassium; **CR:** Creatinine; **U:** Urine; **WCC:** White Cell Count; **GB:** Haemoglobin; **CRP:** C-reactive protein

HFRS with separate laboratory test models had discrimination (sometimes poor, and often fair) AUROC ranging from 0.601-0.762. Whereas HFRS with LDT-EWS had fair and good discrimination for all 9 periods of LOS (0.766-0.813).

Consequently, the models of HFRS with LDT-EWS offered the best values of AUROC compared to models of HFRS with separate laboratory tests.
