# Supplementary material for: Vital signs and common blood tests improve the predictive power of the Hospital Frailty Risk Score to predict poor outcomes across all adult ages
Source: PLoS One. 2026 May 5;21(5):e0348669. doi: 10.1371/journal.pone.0348669 (PMC13143055; doi:10.1371/journal.pone.0348669)
Supplement: S5 Table — Results of AUROC for 9 period of LOS according to admission year. (DOCX) [file pone.0348669.s005.docx]

**S5 Table. (S5a-S5h) Tables. Results of AUROC for 9 period of LOS according to admission year**

**S5a Table. Results of AUROC for 9 period of longer length of stay for Sample data 2012**

| **Outcomes** | **HFRS** | **HFRS + Age** | **HFRS+ Gender** | **HFRS+ LDT-EWS** | **HFRS+ NEWS** | **HFRS + CCI** | **HFRS + CRP** |
| --- | --- | --- | --- | --- | --- | --- | --- |
|  | AUROC | AUROC | AUROC | AUROC | AUROC | AUROC | AUROC |
| **LOS>3-day** | 0.687 | 0.717 | 0.686 | 0.756 | 0.702 | 0.713 | 0.716 |
| **LOS>7-day** | 0.716 | 0.746 | 0.718 | 0.767 | 0.718 | 0.736 | 0.732 |
| **LOS>10-day** | 0.733 | 0.762 | 0.736 | 0.772 | 0.729 | 0.748 | 0.732 |
| **LOS>14-day** | 0.751 | 0.773 | 0.754 | 0.778 | 0.741 | 0.760 | 0.751 |
| **LOS>21-day** | 0.772 | 0.780 | 0.771 | 0.781 | 0.753 | 0.773 | 0.763 |
| **LOS>30-day** | 0.776 | 0.782 | 0.784 | 0.784 | 0.767 | 0.777 | 0.765 |
| **LOS>45-day** | 0.777 | 0.768 | 0.758 | 0.784 | 0.753 | 0.763 | 0.749 |
| **LOS>60-day** | 0.779 | 0.714 | 0.717 | 0.782 | 0.721 | 0.729 | 0.740 |
| **LOS>90-day** | 0.779 | 0.727 | 0.767 | 0.782 | 0.738 | 0.758 | 0.746 |

**HFRS:** Hospital frailty risk score; **NEWS:** aggregate National Early Warning Score; **LDT-EWS:** aggregate Laboratory Decision Tree Early Warning Score; **CCI:** Charlson Comorbidity Index; **CRP:** c-reactive protein test

**S5b Table. Results of AUROC for 9 period of longer length of stay for Sample data 2013**

| **Outcomes** | **HFRS** | **HFRS + Age** | **HFRS+ Gender** | **HFRS+ LDT-EWS** | **HFRS+ NEWS** | **HFRS + CCI** | **HFRS + CRP** |
| --- | --- | --- | --- | --- | --- | --- | --- |
|  | AUROC | AUROC | AUROC | AUROC | AUROC | AUROC | AUROC |
| **LOS>3-day** | 0.707 | 0.742 | 0.707 | 0.761 | 0.711 | 0.732 | 0.723 |
| **LOS>7-day** | 0.739 | 0.771 | 0.740 | 0.777 | 0.731 | 0.76 | 0.740 |
| **LOS>10-day** | 0.749 | 0.777 | 0.749 | 0.782 | 0.74 | 0.764 | 0.744 |
| **LOS>14-day** | 0.760 | 0.780 | 0.760 | 0.784 | 0.745 | 0.770 | 0.748 |
| **LOS>21-day** | 0.780 | 0.788 | 0.780 | 0.790 | 0.758 | 0.782 | 0.76 |
| **LOS>30-day** | 0.788 | 0.788 | 0.789 | 0.791 | 0.762 | 0.783 | 0.765 |
| **LOS>45-day** | 0.800 | 0.778 | 0.799 | 0.804 | 0.779 | 0.796 | 0.769 |
| **LOS>60-day** | 0.791 | 0.749 | 0.789 | 0.794 | 0.774 | 0.786 | 0.769 |
| **LOS>90-day** | 0.807 | 0.780 | 0.793 | 0.826 | 0.790 | 0.805 | 0.788 |

**HFRS:** Hospital frailty risk score; **NEWS:** aggregate National Early Warning Score; **LDT-EWS:** aggregate Laboratory Decision Tree Early Warning Score; **CCI:** Charlson Comorbidity Index; **CRP:** c-reactive protein test

**S5c Table. Results of AUROC for 9 period of longer length of stay for Sample data 2014**

| **Outcomes** | **HFRS** | **HFRS + Age** | **HFRS+ Gender** | **HFRS+ LDT-EWS** | **HFRS+ NEWS** | **HFRS + CCI** | **HFRS + CRP** |
| --- | --- | --- | --- | --- | --- | --- | --- |
|  | AUROC | AUROC | AUROC | AUROC | AUROC | AUROC | AUROC |
| **LOS>3-day** | 0.706 | 0.738 | 0.705 | 0.758 | 0.715 | 0.726 | 0.731 |
| **LOS>7-day** | 0.742 | 0.765 | 0.742 | 0.776 | 0.735 | 0.755 | 0.744 |
| **LOS>10-day** | 0.757 | 0.776 | 0.756 | 0.785 | 0.75 | 0.769 | 0.756 |
| **LOS>14-day** | 0.768 | 0.779 | 0.766 | 0.790 | 0.755 | 0.776 | 0.764 |
| **LOS>21-day** | 0.779 | 0.780 | 0.778 | 0.800 | 0.762 | 0.784 | 0.773 |
| **LOS>30-day** | 0.787 | 0.774 | 0.784 | 0.799 | 0.771 | 0.784 | 0.782 |
| **LOS>45-day** | 0.790 | 0.766 | 0.783 | 0.796 | 0.774 | 0.784 | 0.779 |
| **LOS>60-day** | 0.799 | 0.776 | 0.782 | 0.803 | 0.783 | 0.797 | 0.786 |
| **LOS>90-day** | 0.785 | 0.744 | 0.758 | 0.793 | 0.764 | 0.766 | 0.743 |

**HFRS:** Hospital frailty risk score; **NEWS:** aggregate National Early Warning Score; **LDT-EWS:** aggregate Laboratory Decision Tree Early Warning Score; **CCI:** Charlson Comorbidity Index; **CRP:** c-reactive protein test

**S5d Table. Results of AUROC for 9 period of longer length of stay for Sample data 2015**

| **Outcomes** | **HFRS** | **HFRS + Age** | **HFRS+ Gender** | **HFRS+ LDT-EWS** | **HFRS+ NEWS** | **HFRS + CCI** | **HFRS + CRP** |
| --- | --- | --- | --- | --- | --- | --- | --- |
|  | AUROC | AUROC | AUROC | AUROC | AUROC | AUROC | AUROC |
| **LOS>3-day** | 0.729 | 0.753 | 0.727 | 0.779 | 0.727 | 0.746 | 0.752 |
| **LOS>7-day** | 0.767 | 0.782 | 0.766 | 0.801 | 0.757 | 0.780 | 0.775 |
| **LOS>10-day** | 0.775 | 0.785 | 0.775 | 0.803 | 0.761 | 0.784 | 0.774 |
| **LOS>14-day** | 0.787 | 0.789 | 0.785 | 0.811 | 0.771 | 0.792 | 0.787 |
| **LOS>21-day** | 0.793 | 0.788 | 0.790 | 0.807 | 0.774 | 0.793 | 0.782 |
| **LOS>30-day** | 0.795 | 0.779 | 0.791 | 0.804 | 0.777 | 0.791 | 0.784 |
| **LOS>45-day** | 0.788 | 0.768 | 0.776 | 0.800 | 0.769 | 0.785 | 0.768 |
| **LOS>60-day** | 0.767 | 0.746 | 0.753 | 0.782 | 0.744 | 0.762 | 0.736 |
| **LOS>90-day** | 0.772 | 0.746 | 0.767 | 0.793 | 0.747 | 0.781 | 0.757 |

**HFRS:** Hospital frailty risk score; **NEWS:** aggregate National Early Warning Score; **LDT-EWS:** aggregate Laboratory Decision Tree Early Warning Score; **CCI:** Charlson Comorbidity Index; **CRP:** c-reactive protein test

**S5e Table. Results of AUROC for 9 period of longer length of stay for Sample data 2016**

| **Outcomes** | **HFRS** | **HFRS + Age** | **HFRS+ Gender** | **HFRS+ LDT-EWS** | **HFRS+ NEWS** | **HFRS + CCI** | **HFRS + CRP** |
| --- | --- | --- | --- | --- | --- | --- | --- |
|  | AUROC | AUROC | AUROC | AUROC | AUROC | AUROC | AUROC |
| **LOS>3-day** | 0.716 | 0.739 | 0.716 | 0.765 | 0.711 | 0.720 | 0.726 |
| **LOS>7-day** | 0.750 | 0.768 | 0.750 | 0.779 | 0.735 | 0.752 | 0.743 |
| **LOS>10-day** | 0.756 | 0.770 | 0.76 | 0.780 | 0.739 | 0.756 | 0.742 |
| **LOS>14-day** | 0.763 | 0.774 | 0.763 | 0.781 | 0.744 | 0.761 | 0.749 |
| **LOS>21-day** | 0.776 | 0.780 | 0.774 | 0.788 | 0.753 | 0.773 | 0.759 |
| **LOS>30-day** | 0.786 | 0.775 | 0.780 | 0.795 | 0.760 | 0.784 | 0.773 |
| **LOS>45-day** | 0.776 | 0.761 | 0.768 | 0.786 | 0.750 | 0.792 | 0.778 |
| **LOS>60-day** | 0.784 | 0.773 | 0.783 | 0.794 | 0.760 | 0.790 | 0.789 |
| **LOS>90-day** | 0.782 | 0.753 | 0.772 | 0.810 | 0.761 | 0.807 | 0.775 |

**HFRS:** Hospital frailty risk score; **NEWS:** aggregate National Early Warning Score; **LDT-EWS:** aggregate Laboratory Decision Tree Early Warning Score; **CCI:** Charlson Comorbidity Index; **CRP:** c-reactive protein test

**S5f Table. Results of AUROC for 9 period of longer length of stay for Sample data 2017**

| **Outcomes** | **HFRS** | **HFRS + Age** | **HFRS+ Gender** | **HFRS+ LDT-EWS** | **HFRS+ NEWS** | **HFRS + CCI** | **HFRS + CRP** |
| --- | --- | --- | --- | --- | --- | --- | --- |
|  | AUROC | AUROC | AUROC | AUROC | AUROC | AUROC | AUROC |
| **LOS>3-day** | 0.732 | 0.756 | 0.734 | 0.771 | 0.716 | 0.732 | 0.733 |
| **LOS>7-day** | 0.762 | 0.775 | 0.763 | 0.783 | 0.737 | 0.762 | 0.746 |
| **LOS>10-day** | 0.775 | 0.787 | 0.776 | 0.791 | 0.749 | 0.775 | 0.760 |
| **LOS>14-day** | 0.786 | 0.787 | 0.787 | 0.797 | 0.759 | 0.786 | 0.770 |
| **LOS>21-day** | 0.797 | 0.800 | 0.796 | 0.804 | 0.770 | 0.797 | 0.782 |
| **LOS>30-day** | 0.807 | 0.808 | 0.806 | 0.812 | 0.780 | 0.807 | 0.790 |
| **LOS>45-day** | 0.826 | 0.825 | 0.823 | 0.828 | 0.794 | 0.826 | 0.809 |
| **LOS>60-day** | 0.828 | 0.829 | 0.829 | 0.831 | 0.801 | 0.828 | 0.814 |
| **LOS>90-day** | 0.838 | 0.844 | 0.851 | 0.858 | 0.831 | 0.838 | 0.849 |

**HFRS:** Hospital frailty risk score; **NEWS:** aggregate National Early Warning Score; **LDT-EWS:** aggregate Laboratory Decision Tree Early Warning Score; **CCI:** Charlson Comorbidity Index; **CRP:** c-reactive protein test

**S5g Table. Results of AUROC for 9 period of longer length of stay for Sample data 2018**

| **Outcomes** | **HFRS** | **HFRS + Age** | **HFRS+ Gender** | **HFRS+ LDT-EWS** | **HFRS+ NEWS** | **HFRS + CCI** | **HFRS + CRP** |
| --- | --- | --- | --- | --- | --- | --- | --- |
|  | AUROC | AUROC | AUROC | AUROC | AUROC | AUROC | AUROC |
| **LOS>3-day** | 0.743 | 0.750 | 0.742 | 0.762 | 0.715 | 0.746 | 0.743 |
| **LOS>7-day** | 0.772 | 0.773 | 0.772 | 0.775 | 0.744 | 0.771 | 0.761 |
| **LOS>10-day** | 0.777 | 0.779 | 0.777 | 0.781 | 0.750 | 0.777 | 0.765 |
| **LOS>14-day** | 0.782 | 0.779 | 0.782 | 0.786 | 0.752 | 0.782 | 0.766 |
| **LOS>21-day** | 0.791 | 0.776 | 0.790 | 0.794 | 0.759 | 0.792 | 0.765 |
| **LOS>30-day** | 0.780 | 0.763 | 0.787 | 0.789 | 0.753 | 0.787 | 0.765 |
| **LOS>45-day** | 0.781 | 0.770 | 0.782 | 0.785 | 0.751 | 0.778 | 0.744 |
| **LOS>60-day** | 0.797 | 0.783 | 0.794 | 0.799 | 0.770 | 0.790 | 0.765 |
| **LOS>90-day** | 0.801 | 0.797 | 0.801 | 0.801 | 0.761 | 0.800 | 0.751 |

**HFRS:** Hospital frailty risk score; **NEWS:** aggregate National Early Warning Score; **LDT-EWS:** aggregate Laboratory Decision Tree Early Warning Score; **CCI:** Charlson Comorbidity Index; **CRP:** c-reactive protein test

**S5h Table. Results of AUROC for 9 period of longer length of stay for Sample data 2019**

| **Outcomes** | **HFRS** | **HFRS + Age** | **HFRS+ Gender** | **HFRS+ LDT-EWS** | **HFRS+ NEWS** | **HFRS + CCI** | **HFRS + CRP** |
| --- | --- | --- | --- | --- | --- | --- | --- |
|  | AUROC | AUROC | AUROC | AUROC | AUROC | AUROC | AUROC |
| **LOS>3-day** | 0.760 | 0.763 | 0.758 | 0.766 | 0.731 | 0.765 | 0.748 |
| **LOS>7-day** | 0.790 | 0.786 | 0.790 | 0.794 | 0.760 | 0.793 | 0.767 |
| **LOS>10-day** | 0.801 | 0.797 | 0.800 | 0.809 | 0.775 | 0.803 | 0.779 |
| **LOS>14-day** | 0.809 | 0.797 | 0.808 | 0.813 | 0.777 | 0.805 | 0.780 |
| **LOS>21-day** | 0.811 | 0.800 | 0.810 | 0.817 | 0.782 | 0.808 | 0.780 |
| **LOS>30-day** | 0.811 | 0.798 | 0.810 | 0.817 | 0.783 | 0.808 | 0.782 |
| **LOS>45-day** | 0.806 | 0.788 | 0.806 | 0.812 | 0.7770 | 0.800 | 0.772 |
| **LOS>60-day** | 0.797 | 0.786 | 0.793 | 0.803 | 0.770 | 0.795 | 0.760 |
| **LOS>90-day** | 0.702 | 0.707 | 0.677 | 0.710 | 0.656 | 0.707 | 0.650 |

**HFRS:** Hospital frailty risk score; **NEWS:** aggregate National Early Warning Score; **LDT-EWS:** aggregate Laboratory Decision Tree Early Warning Score; **CCI:** Charlson Comorbidity Index; **CRP:** c-reactive protein test
