# Supplementary material for: Vital signs and common blood tests improve the predictive power of the Hospital Frailty Risk Score to predict poor outcomes across all adult ages
Source: PLoS One. 2026 May 5;21(5):e0348669. doi: 10.1371/journal.pone.0348669 (PMC13143055; doi:10.1371/journal.pone.0348669)
Supplement: S6 Table — Results of AUROC for 9 period of LOS according to age groups. (DOCX) [file pone.0348669.s006.docx]

**S6 Table. (S6a-S6d) Tables. Results of AUROC for 9 period of length of stay according to age groups**

**S6a Table. Results of AUROC for 9 period of longer length of stay for age groups (<45 years)**

| **Outcomes** | **HFRS** | **HFRS + Age** | **HFRS+ Gender** | **HFRS+ LDT-EWS** | **HFRS + NEWS** | **HFRS + CCI** | **HFRS + CRP** |
| --- | --- | --- | --- | --- | --- | --- | --- |
|  | AUROC | AUROC | AUROC | AUROC | AUROC | AUROC | AUROC |
| **LOS>3-day** | 0.674 | 0.680 | 0.677 | **0.741** | 0.662 | 0.680 | 0.715 |
| **LOS>7-day** | 0.726 | 0.731 | 0.726 | **0.788** | 0.707 | 0.734 | 0.749 |
| **LOS>10-day** | 0.753 | 0.745 | 0.747 | **0.815** | 0.738 | 0.759 | 0.767 |
| **LOS>14-day** | 0.779 | 0.769 | 0.764 | **0.836** | 0.760 | 0.786 | 0.789 |
| **LOS>21-day** | 0.815 | 0.804 | 0.799 | **0.861** | 0.785 | 0.819 | 0.816 |
| **LOS>30-day** | 0.823 | 0.812 | 0.811 | **0.865** | 0.792 | 0.822 | 0.824 |
| **LOS>45-day** | 0.854 | 0.804 | 0.833 | **0.877** | 0.820 | 0.850 | 0.817 |
| **LOS>60-day** | 0.870 | 0.843 | 0.848 | **0.876** | 0.837 | 0.869 | 0.834 |
| **LOS>90-day** | 0.894 | 0.875 | 0.894 | **0.902** | 0.877 | 0.890 | 0.884 |

**HFRS:** Hospital frailty risk score; **NEWS:** aggregate National Early Warning Score; **LDT-EWS:** aggregate Laboratory Decision Tree Early Warning Score; **CCI:** Charlson Comorbidity Index; **CRP:** c-reactive protein test

**S6b Table. Results of AUROC for 9 period of longer length of stay for age groups (45-64 years)**

| **Outcomes** | **HFRS** | **HFRS + Age** | **HFRS+ Gender** | **HFRS+ LDT-EWS** | **HFRS + NEWS** | **HFRS + CCI** | **HFRS + CRP** |
| --- | --- | --- | --- | --- | --- | --- | --- |
|  | AUROC | AUROC | AUROC | AUROC | AUROC | AUROC | AUROC |
| **LOS>3-day** | 0.668 | 0.667 | 0.668 | **0.731** | 0.681 | 0.678 | 0.716 |
| **LOS>7-day** | 0.710 | 0.702 | 0.709 | **0.762** | 0.706 | 0.720 | 0.739 |
| **LOS>10-day** | 0.725 | 0.711 | 0.723 | **0.769** | 0.717 | 0.734 | 0.754 |
| **LOS>14-day** | 0.744 | 0.728 | 0.738 | **0.783** | 0.733 | 0.750 | 0.771 |
| **LOS>21-day** | 0.763 | 0.748 | 0.751 | **0.785** | 0.745 | 0.765 | 0.778 |
| **LOS>30-day** | 0.782 | 0.760 | 0.768 | **0.795** | 0.756 | 0.776 | 0.787 |
| **LOS>45-day** | 0.803 | 0.789 | 0.784 | **0.818** | 0.777 | 0.802 | 0.814 |
| **LOS>60-day** | 0.821 | 0.805 | 0.817 | **0.834** | 0.799 | 0.823 | 0.830 |
| **LOS>90-day** | 0.824 | 0.819 | 0.828 | **0.843** | 0.805 | 0.826 | 0.839 |

**HFRS:** Hospital frailty risk score; **NEWS:** aggregate National Early Warning Score; **LDT-EWS:** aggregate Laboratory Decision Tree Early Warning Score; **CCI:** Charlson Comorbidity Index; **CRP:** c-reactive protein test

**S6c Table. Results of AUROC for 9 period of longer length of stay for age groups (65-84 years)**

| **Outcomes** | **HFRS** | **HFRS + Age** | **HFRS+ Gender** | **HFRS+ LDT-EWS** | **HFRS + NEWS** | **HFRS + CCI** | **HFRS + CRP** |
| --- | --- | --- | --- | --- | --- | --- | --- |
|  | AUROC | AUROC | AUROC | AUROC | AUROC | AUROC | AUROC |
| **LOS>3-day** | 0.656 | 0.652 | 0.655 | **0.693** | 0.671 | 0.658 | 0.687 |
| **LOS>7-day** | 0.686 | 0.680 | 0.685 | **0.704** | 0.687 | 0.687 | 0.701 |
| **LOS>10-day** | 0.697 | 0.690 | 0.697 | **0.709** | 0.694 | 0.698 | 0.703 |
| **LOS>14-day** | 0.706 | 0.698 | 0.707 | **0.712** | 0.700 | 0.707 | 0.706 |
| **LOS>21-day** | 0.721 | 0.712 | 0.721 | **0.723** | 0.710 | 0.721 | 0.711 |
| **LOS>30-day** | 0.733 | 0.722 | 0.733 | **0.736** | 0.723 | 0.733 | 0.722 |
| **LOS>45-day** | 0.737 | 0.727 | 0.735 | **0.740** | 0.728 | 0.735 | 0.727 |
| **LOS>60-day** | 0.739 | 0.735 | 0.737 | **0.743** | 0.732 | 0.739 | 0.734 |
| **LOS>90-day** | 0.747 | 0.751 | 0.742 | **0.751** | 0.740 | 0.743 | 0.724 |

**HFRS:** Hospital frailty risk score; **NEWS:** aggregate National Early Warning Score; **LDT-EWS:** aggregate Laboratory Decision Tree Early Warning Score; **CCI:** Charlson Comorbidity Index; **CRP:** c-reactive protein test

**S6d Table. Results of AUROC for 9 period of longer length of stay for age groups (≥85 years)**

| **Outcomes** | **HFRS** | **HFRS + Age** | **HFRS+ Gender** | **HFRS+ LDT-EWS** | **HFRS + NEWS** | **HFRS + CCI** | **HFRS + CRP** |
| --- | --- | --- | --- | --- | --- | --- | --- |
|  | AUROC | AUROC | AUROC | AUROC | AUROC | AUROC | AUROC |
| **LOS>3-day** | 0.616 | 0.615 | 0.615 | **0.653** | 0.626 | 0.622 | 0.654 |
| **LOS>7-day** | 0.626 | 0.626 | 0.626 | **0.643** | 0.63 | 0.628 | 0.645 |
| **LOS>10-day** | 0.629 | 0.629 | 0.628 | **0.639** | 0.63 | 0.630 | 0.636 |
| **LOS>14-day** | 0.632 | 0.633 | 0.633 | **0.636** | 0.629 | 0.633 | 0.635 |
| **LOS>21-day** | 0.637 | 0.637 | 0.637 | **0.640** | 0.630 | 0.635 | 0.630 |
| **LOS>30-day** | 0.642 | 0.643 | 0.642 | **0.644** | 0.636 | 0.643 | 0.635 |
| **LOS>45-day** | 0.635 | 0.628 | 0.633 | **0.639** | 0.627 | 0.637 | 0.612 |
| **LOS>60-day** | 0.623 | 0.615 | 0.617 | **0.647** | 0.616 | 0.643 | 0.608 |
| **LOS>90-day** | 0.647 | 0.655 | 0.623 | **0.652** | 0.639 | 0.643 | 0.627 |

**HFRS:** Hospital frailty risk score; **NEWS:** aggregate National Early Warning Score; **LDT-EWS:** aggregate Laboratory Decision Tree Early Warning Score; **CCI:** Charlson Comorbidity Index; **CRP:** c-reactive protein test
