# Supplementary material for: Vital signs and common blood tests improve the predictive power of the Hospital Frailty Risk Score to predict poor outcomes across all adult ages
Source: PLoS One. 2026 May 5;21(5):e0348669. doi: 10.1371/journal.pone.0348669 (PMC13143055; doi:10.1371/journal.pone.0348669)
Supplement: S7 Table — Results of AUROC for 9 period of LOS according to gender. (DOCX) [file pone.0348669.s007.docx]

**S7 Table. (S7a-S7b) Tables. Results of AUROC curve for 9 period of longer length of stay according to gender**

**S7a Table. Results of AUROC for 9 period of longer length of stay for males**

| **Outcomes** | **HFRS** | **HFRS + Age** | **HFRS+ LDT-EWS** | **HFRS + NEWS** | **HFRS + CCI** | **HFRS + CRP** |
| --- | --- | --- | --- | --- | --- | --- |
|  | AUROC | AUROC | AUROC | AUROC | AUROC | AUROC |
| **LOS>3-day** | 0.739 | 0.766 | 0.778 | 0.720 | 0.751 | 0.748 |
| **LOS>7-day** | 0.768 | 0.789 | 0.790 | 0.741 | 0.777 | 0.761 |
| **LOS>10-day** | 0.777 | 0.795 | 0.793 | 0.747 | 0.784 | 0.766 |
| **LOS>14-day** | 0.780 | 0.795 | 0.791 | 0.749 | 0.786 | 0.767 |
| **LOS>21-day** | 0.787 | 0.792 | 0.793 | 0.751 | 0.790 | 0.765 |
| **LOS>30-day** | 0.793 | 0.788 | 0.795 | 0.757 | 0.793 | 0.766 |
| **LOS>45-day** | 0.793 | 0.780 | 0.795 | 0.753 | 0.791 | 0.760 |
| **LOS>60-day** | 0.789 | 0.774 | 0.790 | 0.751 | 0.780 | 0.761 |
| **LOS>90-day** | 0.809 | 0.777 | 0.809 | 0.769 | 0.803 | 0.765 |

**HFRS:** Hospital frailty risk score; **NEWS:** aggregate National Early Warning Score; **LDT-EWS:** aggregate Laboratory Decision Tree Early Warning Score; **CCI:** Charlson Comorbidity Index; **CRP:** c-reactive protein test

**S7b Table. Results of AUROC for 9 period of longer length of stay for females**

| **Outcomes** | **HFRS** | **HFRS + Age** | **HFRS+ LDT-EWS** | **HFRS + NEWS** | **HFRS + CCI** | **HFRS + CRP** |
| --- | --- | --- | --- | --- | --- | --- |
|  | AUROC | AUROC | AUROC | AUROC | AUROC | AUROC |
| **LOS>3-day** | 0.701 | 0.716 | 0.745 | 0.711 | 0.711 | 0.725 |
| **LOS>7-day** | 0.736 | 0.743 | 0.761 | 0.732 | 0.742 | 0.741 |
| **LOS>10-day** | 0.748 | 0.751 | 0.766 | 0.740 | 0.752 | 0.744 |
| **LOS>14-day** | 0.762 | 0.759 | 0.772 | 0.750 | 0.763 | 0.752 |
| **LOS>21-day** | 0.774 | 0.765 | 0.775 | 0.757 | 0.774 | 0.756 |
| **LOS>30-day** | 0.780 | 0.763 | 0.781 | 0.761 | 0.779 | 0.766 |
| **LOS>45-day** | 0.781 | 0.759 | 0.782 | 0.763 | 0.781 | 0.764 |
| **LOS>60-day** | 0.781 | 0.752 | 0.782 | 0.766 | 0.782 | 0.768 |
| **LOS>90-day** | 0.764 | 0.738 | 0.764 | 0.749 | 0.764 | 0.754 |

**HFRS:** Hospital frailty risk score; **NEWS:** aggregate National Early Warning Score; **LDT-EWS:** aggregate Laboratory Decision Tree Early Warning Score; **CCI:** Charlson Comorbidity Index; **CRP:** c-reactive protein test
