# Supplementary material for: Vital signs and common blood tests improve the predictive power of the Hospital Frailty Risk Score to predict poor outcomes across all adult ages
Source: PLoS One. 2026 May 5;21(5):e0348669. doi: 10.1371/journal.pone.0348669 (PMC13143055; doi:10.1371/journal.pone.0348669)
Supplement: S8 Table — (DOCX) [file pone.0348669.s008.docx]

**S8 Table. Results of AUROC for 9 periods of longer length of stay for multivariate models.**

| **Outcomes** | **HFRS alone** | **HFRS+LDT-EWS** | **Multivariate model** |
| --- | --- | --- | --- |
|  | AUROC  (95% CI) | AUROC  (95% CI) | AUROC  (95% CI) |
| **LOS>3-day** | 0.723 | **0.764** | 0.673 |
|  | (0.719-0.726) | **(0.762-0.767)** | (0.668-0.677) |
| **LOS>7-day** | 0.757 | **0.782** | 0.688 |
|  | (0.754-0.761) | **(0.778-0.785)** | (0.683-0.693) |
| **LOS>10-day** | 0.770 | **0.786** | 0.712 |
|  | (0.766-0.773) | **(0.783-0.79)** | (0.706-0.717) |
| **LOS>14-day** | 0.779 | **0.789** | 0.713 |
|  | (0.775-0.783) | **(0.786-0.793)** | (0.707-0.719) |
| **LOS>21-day** | 0.789 | **0.793** | 0.708 |
|  | (0.784-0.794) | **(0.788-0.797)** | (0.701-0.716) |
| **LOS>30-day** | 0.796 | **0.798** | 0.697 |
|  | (0.790-0.801) | **(0.787-0.799)** | (0.687-0.707) |
| **LOS>45-day** | 0.798 | **0.805** | 0.678 |
|  | (0.790-0.805) | **(0.786-0.808)** | (0.664-0.692) |
| **LOS>60-day** | 0.797 | **0.810** | 0.654 |
|  | (0.786-0.807) | **(0.779-0.815)** | (0.634-0.674) |
| **LOS>90-day** | 0.798 | **0.810** | 0.636 |
|  | (0.781-0.816) | **(0.773-0.809)** | (0.595-0.676) |

**Multivariate model:** HFRS+ age+ gender+ LDT-EWS+ NEWS+ CCI+ CRP

**HFRS:** Hospital frailty risk score; **NEWS:** aggregate National Early Warning Score; **LDT-EWS:** aggregate Laboratory Decision Tree Early Warning Score; **CCI:** Charlson Comorbidity Index; **CRP:** c-reactive protein test
