# Supplementary material for: Vital signs and common blood tests improve the predictive power of the Hospital Frailty Risk Score to predict poor outcomes across all adult ages
Source: PLoS One. 2026 May 5;21(5):e0348669. doi: 10.1371/journal.pone.0348669 (PMC13143055; doi:10.1371/journal.pone.0348669)
Supplement: S9 Table — (DOCX) [file pone.0348669.s009.docx]

**S9 Table. Results of AUROC for 9 periods of length of stay for data after included patients who died in hospita****l** **for each variable alone and HFRS combined with one other variable**

| **Outcomes** | **LOS>3-day** | **LOS>7-day** | **LOS>10-day** | **LOS>14-day** | **LOS>21-day** | **LOS>30-day** | **LOS>45-day** | **LOS>60-day** | **LOS>90-day** |
| --- | --- | --- | --- | --- | --- | --- | --- | --- | --- |
|  | AUROC  (95% CI) | AUROC  (95% CI) | AUROC  (95% CI) | AUROC  (95% CI) | AUROC  (95% CI) | AUROC  (95% CI) | AUROC  (95% CI) | AUROC  (95% CI) | AUROC  (95% CI) |
| **HFRS alone** | 0.725 | 0.757 | 0.767 | 0.775 | 0.784 | 0.789 | 0.789 | 0.790 | 0.790 |
|  | (0.722-0.728) | (0.753-0.76) | (0.764-0.771) | (0.771-0.779) | (0.78-0.789) | (0.785-0.796) | (0.783-0.798) | (0.778-0.798) | (0.772-0.807) |
| **Age alone** | 0.714 | 0.733 | 0.738 | 0.738 | 0.734 | 0.720 | 0.704 | 0.682 | 0.624 |
|  | (0.711-0.717) | (0.729-0.736) | (0.734-0.741) | (0.734-0.742) | (0.729-0.739) | (0.714-0.726) | (0.695-0.713) | (0.668-0.695) | (0.598-0.649) |
| **Gender alone** | 0.519 | 0.509 | 0.506 | 0.505 | 0.505 | 0.509 | 0.521 | 0.523 | 0.533 |
|  | (0.516-0.522) | (0.505-0.512) | (0.503-0.51) | (0.501-0.51) | (0.499-0.51) | (0.502-0.517) | (0.511-0.532) | (0.509-0.538) | (0.506-0.56) |
| **LDT-EWS alone** | 0.725 | 0.718 | 0.712 | 0.703 | 0.690 | 0.679 | 0.674 | 0.659 | 0.660 |
|  | (0.723-0.729) | (0.715-0.722) | (0.708-0.716) | (0.699-0.708) | (0.684-0.695) | (0.671-0.686) | (0.663-0.685) | (0.644-0.674) | (0.632-0.687) |
| **NEWS alone** | 0.617 | 0.606 | 0.600 | 0.596 | 0.586 | 0.583 | 0.565 | 0.564 | 0.581 |
|  | (0.613-0.62) | (0.601-0.61) | (0.596-0.605) | (0.59-0.601) | (0.579-0.592) | (0.574-0.591) | (0.553-0.577) | (0.547-0.581) | (0.551-0.612) |
| **CCI alone** | 0.617 | 0.619 | 0.618 | 0.612 | 0.604 | 0.582 | 0.563 | 0.536 | 0.505 |
|  | (0.614-0.62) | (0.615-0.623) | (0.614-0.622) | (0.607-0.617) | (0.597-0.61) | (0.574-0.59) | (0.552-0.575) | (0.52-0.552) | (0.477-0.533) |
| **CRP alone** | 0.667 | 0.647 | 0.637 | 0.628 | 0.619 | 0.616 | 0.605 | 0.611 | 0.619 |
|  | (0.663-0.672) | (0.642-0.652) | (0.631-0.642) | (0.621-0.634) | (0.611-0.627) | (0.606-0.627) | (0.59-0.62) | (0.589-0.632) | (0.578-0.66) |
| **HFRS+ age** | 0.747 | 0.772 | 0.779 | 0.780 | 0.783 | 0.779 | 0.773 | 0.767 | 0.758 |
|  | (0.744-0.75) | (0.769-0.775) | (0.776-0.782) | (0.778-0.786) | (0.779-0.788) | (0.773-0.785) | (0.765-0.781) | (0.755-0.778) | (0.736-0.779) |
| **HFRS+ gender** | 0.724 | 0.756 | 0.767 | 0.774 | 0.783 | 0.788 | 0.783 | 0.778 | 0.778 |
|  | (0.721-0.727) | (0.753-0.76) | (0.764-0.771) | (0.77-0.778) | (0.778-0.787) | (0.782-0.793) | (0.775-0.791) | (0.767-0.789) | (0.758-0.797) |
| **HFRS+ LDT-EWS** | **0.766** | **0.780** | **0.784** | **0.787** | **0.791** | **0.800** | **0.807** | **0.813** | **0.813** |
|  | **(0.763-0.769)** | **(0.777-0.783)** | **(0.78-0.787)** | **(0.782-0.789)** | **(0.783-0.796)** | **(0.782-0.805)** | **(0.78-0.820)** | **(0.773-0.821)** | **(0.766-0.821)** |
| **HFRS+ NEWS** | 0.719 | 0.740 | 0.747 | 0.752 | 0.757 | 0.762 | 0.760 | 0.761 | 0.761 |
|  | (0.715-0.722) | (0.736-0.743) | (0.743-0.751) | (0.748-0.757) | (0.752-0.762) | (0.756-0.768) | (0.752-0.769) | (0.75-0.772) | (0.743-0.78) |
| **HFRS+ CCI** | 0.737 | 0.764 | 0.773 | 0.779 | 0.786 | 0.787 | 0.788 | 0.787 | 0.788 |
|  | (0.734-0.74) | (0.761-0.767) | (0.769-0.776) | (0.775-0.782) | (0.782-0.79) | (0.784-0.795) | (0.783-0.798) | (0.776-0.797) | (0.772-0.809) |
| **HFRS+CRP** | 0.740 | 0.755 | 0.759 | 0.763 | 0.764 | 0.769 | 0.764 | 0.767 | 0.762 |
|  | (0.736-0.744) | (0.751-0.759) | (0.754-0.764) | (0.758-0.768) | (0.758-0.77) | (0.761-0.776) | (0.754-0.775) | (0.752-0.781) | (0.737-0.788) |

**HFRS:** Hospital frailty risk score; **NEWS:** aggregate National Early Warning Score; **LDT-EWS:** aggregate Laboratory Decision Tree Early Warning Score; **CCI:** Charlson Comorbidity Index; **CRP:** c-reactive protein test
