# Supplementary material for: Vital signs and common blood tests improve the predictive power of the Hospital Frailty Risk Score to predict poor outcomes across all adult ages
Source: PLoS One. 2026 May 5;21(5):e0348669. doi: 10.1371/journal.pone.0348669 (PMC13143055; doi:10.1371/journal.pone.0348669)
Supplement: S10 Table — (DOCX) [file pone.0348669.s010.docx]

**S10 Table. AUROC for 8 prediction in-hospital mortality for each variable alone and HFRS combined with one other variable (n=378,916)**

|  | 3 days-mortality (n=5315 death) | 7 days-mortality (n=8542 death) | 10days-mortality (n=10175 death) | 14days-mortality (n=11770 death) | 30days-mortality (n=14847 death) | 60days-mortality (n=16123 death) | 90days-mortality (n=16376 death) | 6 month-mortality (n=16460 death) |
| --- | --- | --- | --- | --- | --- | --- | --- | --- |
|  | AUROC (95%CI) | AUROC (95%CI) | AUROC (95%CI) | AUROC (95%CI) | AUROC (95%CI) | AUROC (95%CI) | AUROC (95%CI) | AUROC (95%CI) |
| **HFRS alone** | 0.648 | 0.669 | 0.679 | 0.688 | 0.705 | 0.714 | 0.716 | 0.716 |
|  | (0.636-0.661) | (0.66-0.679) | (0.67-0.687) | (0.68-0.696) | (0.699-0.712) | (0.708-0.721) | (0.709-0.722) | (0.71-0.723) |
| **Age alone** | 0.734 | 0.739 | 0.741 | 0.743 | 0.750 | 0.754 | 0.754 | 0.754 |
|  | (0.724-0.744) | (0.731-0.746) | (0.734-0.748) | (0.736-0.749) | (0.744-0.756) | (0.748-0.76) | (0.749-0.76) | (0.748-0.759) |
| **Gender alone** | 0.517 | 0.521 | 0.519 | 0.521 | 0.525 | 0.526 | 0.527 | 0.527 |
|  | (0.505-0.53) | (0.511-0.53) | (0.51-0.528) | (0.513-0.53) | (0.518-0.533) | (0.519-0.533) | (0.52-0.534) | (0.52-0.534) |
| **LDT-EWS alone** | 0.730 | 0.723 | 0.725 | 0.728 | 0.730 | 0.731 | 0.730 | 0.730 |
|  | (0.718-0.743) | (0.716-0.736) | (0.716-0.735) | (0.719-0.736) | (0.722-0.737) | (0.723-0.738) | (0.723-0.737) | (0.722-0.737) |
| **NEWS alone** | 0.815 | 0.790 | 0.782 | 0.767 | 0.747 | 0.742 | 0.740 | 0.738 |
|  | (0.803-0.828) | (0.78-0.801) | (0.773-0.792) | (0.758-0.776) | (0.739-0.755) | (0.734-0.75) | (0.732-0.747) | (0.73-0.746) |
| **CCI alone** | 0.650 | 0.657 | 0.658 | 0.662 | 0.662 | 0.660 | 0.659 | 0.659 |
|  | (0.643-0.671) | (0.647-0.668) | (0.648-0.669) | (0.652-0.671) | (0.654-0.671) | (0.652-0.668) | (0.651-0.667) | (0.65-0.667) |
| **CRP alone** | 0.707 | 0.714 | 0.719 | 0.720 | 0.718 | 0.715 | 0.714 | 0.714 |
|  | (0.699-0.732) | (0.701-0.727) | (0.707-0.73) | (0.709-0.731) | (0.709-0.727) | (0.706-0.724) | (0.705-0.723) | (0.705-0.723) |
| **HFRS+Age** | 0.734 | 0.740 | 0.742 | 0.745 | 0.755 | 0.761 | 0.762 | 0.762 |
|  | (0.724-0.744) | (0.732-0.748) | (0.735-0.749) | (0.739-0.752) | (0.75-0.761) | (0.756-0.766) | (0.757-0.767) | (0.756-0.767) |
| **HFRS+Gender** | 0.633 | 0.653 | 0.660 | 0.669 | 0.690 | 0.701 | 0.702 | 0.702 |
|  | (0.62-0.646) | (0.643-0.663) | (0.651-0.669) | (0.661-0.677) | (0.683-0.697) | (0.694-0.707) | (0.695-0.709) | (0.696-0.709) |
| **HFRS+LDT_EWS** | 0.757 | 0.772 | 0.774 | 0.778 | **0.789** | **0.790** | **0.790** | **0.794** |
|  | (0.745-0.769) | (0.763-0.78) | (0.767-0.782) | (0.771-0.785) | **(0.78-0.792)** | **(0.783-0.794)** | **(0.784-0.795)** | **(0.784-0.797)** |
| **HFRS+NEWS** | **0.829** | **0.809** | **0.799** | **0.786** | 0.780 | 0.779 | 0.778 | 0.778 |
|  | **(0.817-0.841)** | **(0.8-0.819)** | **(0.79-0.807)** | **(0.780-0.796)** | (0.773-0.787) | (0.773-0.786) | (0.772-0.785) | (0.772-0.785) |
| **HFRS+CCI** | 0.704 | 0.721 | 0.727 | 0.733 | 0.745 | 0.750 | 0.751 | 0.751 |
|  | (0.692-0.717) | (0.712-0.73) | (0.719-0.735) | (0.725-0.74) | (0.738-0.751) | (0.744-0.756) | (0.745-0.757) | (0.745-0.757) |
| **HFRS+CRP** | 0.708 | 0.720 | 0.723 | 0.727 | 0.736 | 0.739 | 0.740 | 0.741 |
|  | (0.691-0.725) | (0.707-0.733) | (0.712-0.735) | (0.716-0.737) | (0.727-0.745) | (0.731-0.748) | (0.732-0.749) | (0.733-0.749) |

**HFRS:** Hospital frailty risk score; **NEWS2:** aggregate National Early Warning Score2; **LDT‑EWS:** aggregate Laboratory Decision Tree Early Warning Score; **CCI:** Charlson Comorbidity Index; **CRP:** c-reactive protein test.
