# Supplementary material for: Vital signs and common blood tests improve the predictive power of the Hospital Frailty Risk Score to predict poor outcomes across all adult ages
Source: PLoS One. 2026 May 5;21(5):e0348669. doi: 10.1371/journal.pone.0348669 (PMC13143055; doi:10.1371/journal.pone.0348669)
Supplement: S11 Table — (DOCX) [file pone.0348669.s011.docx]

**S11 Table: AUROC for HFRS combined with one other variable according to admission year for 8 periods of in-hospital mortality**

| **2012 data (n=46642)** | | | | | | | | |
| --- | --- | --- | --- | --- | --- | --- | --- | --- |
|  | **3days-mortality** | **7days-mortality** | **10days-mortality** | **14days-mortality** | **30days-mortality** | **60days-mortality** | **90days-mortality** | **6 month-mortality** |
|  | **AUROC (95%CI)** | **AUROC (95%CI)** | **AUROC (95%CI)** | **AUROC (95%CI)** | **AUROC (95%CI)** | **AUROC (95%CI)** | **AUROC (95%CI)** | **AUROC (95%CI)** |
| **HFRS** | 0.624 | 0.654 | 0.659 | 0.664 | 0.682 | 0.69 | 0.688 | 0.689 |
|  | (0.589-0.659) | (0.628-0.679) | (0.635-0.683) | (0.643-0.686) | (0.663-0.702) | (0.671-0.708) | (0.67-0.707) | (0.67-0.707) |
| **HFRS+Age** | 0.76 | 0.765 | 0.766 | 0.762 | 0.771 | 0.775 | 0.775 | 0.775 |
|  | (0.736-0.784) | (0.746-0.784) | (0.749-0.784) | (0.746-0.779) | (0.757-0.786) | (0.761-0.789) | (0.761-0.789) | (0.761-0.789) |
| **HFRS+Gender** | 0.628 | 0.647 | 0.653 | 0.657 | 0.675 | 0.682 | 0.679 | 0.679 |
|  | (0.594-0.663) | (0.621-0.673) | (0.629-0.677) | (0.634-0.679) | (0.655-0.695) | (0.662-0.701) | (0.659-0.698) | (0.66-0.698) |
| **HFRS+LDT-EWS** | 0.766 | 0.792 | 0.793 | 0.797 | **0.805** | **0.804** | **0.803** | **0.803** |
|  | (0.736-0.796) | (0.771-0.813) | (0.774-0.812) | (0.779-0.815) | **(0.789-0.82)** | **(0.789-0.819)** | **(0.788-0.818)** | **(0.788-0.818)** |
| **HFRS+NEWS** | **0.837** | **0.821** | **0.813** | **0.802** | 0.786 | 0.783 | 0.783 | 0.783 |
|  | **(0.807-0.868)** | **(0.798-0.845)** | **(0.791-0.835)** | **(0.782-0.823)** | (0.767-0.805) | (0.765-0.802) | (0.765-0.801) | (0.765-0.801) |
| **HFRS+Charlson(CCI)** | 0.738 | 0.755 | 0.757 | 0.759 | 0.762 | 0.762 | 0.761 | 0.760 |
|  | (0.705-0.771) | (0.731-0.779) | (0.735-0.78) | (0.738-0.779) | (0.744-0.781) | (0.744-0.779) | (0.743-0.778) | (0.742-0.778) |
| **HFRS+CRP** | 0.656 | 0.69 | 0.698 | 0.703 | 0.719 | 0.722 | 0.721 | 0.721 |
|  | (0.6-0.711) | (0.652-0.729) | (0.664-0.733) | (0.67-0.735) | (0.691-0.747) | (0.696-0.749) | (0.695-0.748) | (0.695-0.748) |
| **2013 data (n=45922)** | | | | | | | | |
|  | **3days-mortality** | **7days-mortality** | **10days-mortality** | **14days-mortality** | **30days-mortality** | **60days-mortality** | **90days-mortality** | **6 month-mortality** |
|  | **AUROC (95%CI)** | **AUROC (95%CI)** | **AUROC (95%CI)** | **AUROC (95%CI)** | **AUROC (95%CI)** | **AUROC (95%CI)** | **AUROC (95%CI)** | **AUROC (95%CI)** |
| **HFRS** | 0.679 | 0.688 | 0.694 | 0.695 | 0.704 | 0.714 | 0.714 | 0.715 |
|  | (0.646-0.712) | (0.662-0.713) | (0.671-0.716) | (0.674-0.716) | (0.685-0.723) | (0.696-0.732) | (0.697-0.732) | (0.698-0.733) |
| **HFRS+Age** | 0.744 | 0.753 | 0.755 | 0.751 | 0.761 | 0.768 | 0.768 | 0.767 |
|  | (0.717-0.772) | (0.732-0.773) | (0.737-0.774) | (0.733-0.768) | (0.746-0.776) | (0.754-0.783) | (0.754-0.783) | (0.753-0.782) |
| **HFRS+Gender** | 0.657 | 0.670 | 0.670 | 0.670 | 0.682 | 0.697 | 0.695 | 0.697 |
|  | (0.621-0.693) | (0.642-0.697) | (0.644-0.695) | (0.646-0.694) | (0.661-0.703) | (0.677-0.716) | (0.676-0.715) | (0.677-0.716) |
| **HFRS+LDT-EWS** | 0.788 | 0.785 | 0.791 | 0.796 | **0.800** | **0.805** | **0.805** | **0.806** |
|  | (0.758-0.817) | (0.763-0.808) | (0.771-0.811) | (0.778-0.815) | **(0.785-0.816)** | **(0.79-0.82)** | **(0.791-0.82)** | **(0.791-0.821)** |
| **HFRS+NEWS** | **0.848** | **0.820** | **0.811** | **0.798** | 0.791 | 0.785 | 0.784 | 0.784 |
|  | **(0.818-0.879)** | **(0.795-0.845)** | **(0.789-0.834)** | **(0.776-0.819)** | (0.772-0.81) | (0.767-0.803) | (0.766-0.802) | (0.766-0.802) |
| **HFRS+Charlson(CCI)** | 0.717 | 0.733 | 0.738 | 0.752 | 0.768 | 0.771 | 0.772 | 0.771 |
|  | (0.683-0.751) | (0.708-0.759) | (0.715-0.761) | (0.731-0.773) | (0.75-0.787) | (0.753-0.788) | (0.754-0.789) | (0.754-0.788) |
| **HFRS+CRP** | 0.801 | 0.791 | 0.778 | 0.777 | 0.762 | 0.763 | 0.763 | 0.765 |
|  | (0.763-0.84) | (0.76-0.822) | (0.749-0.807) | (0.751-0.804) | (0.738-0.786) | (0.74-0.786) | (0.74-0.785) | (0.742-0.787) |
| **2014 data (n=45668)** | | | | | | | | |
|  | **3days-mortality** | **7days-mortality** | **10days-mortality** | **14days-mortality** | **30days-mortality** | **60days-mortality** | **90days-mortality** | **6 month-mortality** |
|  | **AUROC (95%CI)** | **AUROC (95%CI)** | **AUROC (95%CI)** | **AUROC (95%CI)** | **AUROC (95%CI)** | **AUROC (95%CI)** | **AUROC (95%CI)** | **AUROC (95%CI)** |
| **HFRS** | 0.640 | 0.653 | 0.666 | 0.674 | 0.695 | 0.703 | 0.706 | 0.706 |
|  | (0.605-0.676) | (0.625-0.681) | (0.64-0.691) | (0.652-0.697) | (0.675-0.716) | (0.683-0.722) | (0.686-0.725) | (0.687-0.725) |
| **HFRS+Age** | 0.733 | 0.736 | 0.742 | 0.746 | 0.754 | 0.754 | 0.753 | 0.752 |
|  | (0.705-0.762) | (0.714-0.759) | (0.722-0.763) | (0.727-0.765) | (0.737-0.77) | (0.738-0.77) | (0.737-0.769) | (0.737-0.768) |
| **HFRS+Gender** | 0.638 | 0.654 | 0.664 | 0.670 | 0.686 | 0.695 | 0.697 | 0.698 |
|  | (0.602-0.674) | (0.626-0.682) | (0.638-0.689) | (0.646-0.693) | (0.665-0.708) | (0.674-0.716) | (0.677-0.718) | (0.678-0.718) |
| **HFRS+LDT-EWS** | 0.766 | 0.775 | 0.778 | 0.783 | **0.793** | **0.799** | **0.800** | **0.800** |
|  | (0.732-0.799) | (0.75-0.8) | (0.756-0.801) | (0.763-0.803) | **(0.776-0.811)** | **(0.782-0.815)** | **(0.784-0.817)** | **(0.784-0.816)** |
| **HFRS+NEWS** | **0.847** | **0.829** | **0.812** | **0.793** | 0.785 | 0.781 | 0.781 | 0.781 |
|  | **(0.811-0.883)** | **(0.801-0.856)** | **(0.786-0.838)** | **(0.769-0.818)** | (0.764-0.806) | (0.761-0.801) | (0.761-0.8) | (0.761-0.8) |
| **HFRS+Charlson(CCI)** | 0.738 | 0.741 | 0.748 | 0.749 | 0.763 | 0.768 | 0.769 | 0.769 |
|  | (0.707-0.77) | (0.716-0.766) | (0.725-0.772) | (0.728-0.771) | (0.744-0.783) | (0.749-0.786) | (0.751-0.787) | (0.751-0.787) |
| **HFRS+CRP** | 0.709 | 0.726 | 0.725 | 0.725 | 0.747 | 0.751 | 0.751 | 0.750 |
|  | (0.657-0.762) | (0.688-0.765) | (0.691-0.76) | (0.693-0.757) | (0.719-0.775) | (0.725-0.778) | (0.725-0.777) | (0.724-0.776) |
| **2015 data (n=45745)** | | | | | | | | |
|  | **3days-mortality** | **7days-mortality** | **10days-mortality** | **14days-mortality** | **30days-mortality** | **60days-mortality** | **90days-mortality** | **6 month-mortality** |
|  | **AUROC (95%CI)** | **AUROC (95%CI)** | **AUROC (95%CI)** | **AUROC (95%CI)** | **AUROC (95%CI)** | **AUROC (95%CI)** | **AUROC (95%CI)** | **AUROC (95%CI)** |
| **HFRS** | 0.624 | 0.659 | 0.668 | 0.680 | 0.702 | 0.711 | 0.712 | 0.712 |
|  | (0.592-0.657) | (0.634-0.684) | (0.645-0.691) | (0.659-0.701) | (0.684-0.721) | (0.693-0.729) | (0.694-0.729) | (0.695-0.73) |
| **HFRS+Age** | 0.718 | 0.721 | 0.732 | 0.739 | 0.750 | 0.754 | 0.754 | 0.754 |
|  | (0.689-0.747) | (0.699-0.743) | (0.712-0.752) | (0.721-0.758) | (0.733-0.766) | (0.739-0.77) | (0.739-0.77) | (0.739-0.77) |
| **HFRS+Gender** | 0.610 | 0.626 | 0.634 | 0.648 | 0.682 | 0.693 | 0.694 | 0.694 |
|  | (0.577-0.643) | (0.599-0.653) | (0.609-0.659) | (0.625-0.671) | (0.662-0.702) | (0.673-0.712) | (0.675-0.713) | (0.675-0.713) |
| **HFRS+LDT-EWS** | 0.766 | 0.779 | 0.782 | 0.787 | **0.798** | **0.798** | **0.799** | **0.799** |
|  | (0.734-0.798) | (0.756-0.802) | (0.762-0.802) | (0.768-0.806) | **(0.782-0.814)** | **(0.783-0.814)** | **(0.783-0.814)** | **(0.784-0.814)** |
| **HFRS+NEWS** | **0.806** | **0.804** | **0.796** | **0.789** | 0.778 | 0.781 | 0.779 | 0.779 |
|  | **(0.762-0.85)** | **(0.777-0.832)** | **(0.771-0.82)** | **(0.766-0.813)** | (0.758-0.798) | (0.762-0.799) | (0.761-0.797) | (0.761-0.798) |
| **HFRS+Charlson(CCI)** | 0.726 | 0.746 | 0.754 | 0.763 | 0.774 | 0.778 | 0.778 | 0.778 |
|  | (0.694-0.758) | (0.721-0.77) | (0.731-0.776) | (0.743-0.783) | (0.757-0.792) | (0.761-0.795) | (0.762-0.795) | (0.762-0.795) |
| **HFRS+CRP** | 0.694 | 0.715 | 0.715 | 0.717 | 0.724 | 0.730 | 0.732 | 0.733 |
|  | (0.644-0.745) | (0.676-0.753) | (0.681-0.75) | (0.686-0.749) | (0.698-0.751) | (0.704-0.755) | (0.707-0.757) | (0.707-0.758) |

| **2016 data (n=45973)** | | | | | | | | |
| --- | --- | --- | --- | --- | --- | --- | --- | --- |
|  | **3days-mortality** | **7days-mortality** | **10days-mortality** | **14days-mortality** | **30days-mortality** | **60days-mortality** | **90days-mortality** | **6 month-mortality** |
|  | **AUROC (95%CI)** | **AUROC (95%CI)** | **AUROC (95%CI)** | **AUROC (95%CI)** | **AUROC (95%CI)** | **AUROC (95%CI)** | **AUROC (95%CI)** | **AUROC (95%CI)** |
| **HFRS** | 0.654 | 0.676 | 0.689 | 0.695 | 0.708 | 0.719 | 0.722 | 0.722 |
|  | (0.616-0.691) | (0.649-0.704) | (0.665-0.713) | (0.673-0.717) | (0.689-0.727) | (0.701-0.737) | (0.705-0.74) | (0.704-0.74) |
| **HFRS+Age** | 0.723 | 0.741 | 0.744 | 0.746 | 0.751 | 0.756 | 0.759 | 0.757 |
|  | (0.692-0.753) | (0.719-0.763) | (0.725-0.764) | (0.728-0.764) | (0.735-0.766) | (0.741-0.771) | (0.744-0.774) | (0.742-0.772) |
| **HFRS+Gender** | 0.637 | 0.661 | 0.667 | 0.669 | 0.689 | 0.701 | 0.704 | 0.704 |
|  | (0.597-0.676) | (0.632-0.69) | (0.641-0.692) | (0.646-0.693) | (0.669-0.71) | (0.681-0.72) | (0.685-0.723) | (0.685-0.723) |
| **HFRS+LDT-EWS** | 0.753 | 0.774 | 0.782 | 0.781 | **0.790** | **0.795** | **0.796** | **0.795** |
|  | (0.719-0.787) | (0.75-0.797) | (0.761-0.803) | (0.764-0.802) | **(0.774-0.807)** | **(0.78-0.81)** | **(0.781-0.811)** | **(0.781-0.81)** |
| **HFRS+NEWS** | **0.845** | **0.823** | **0.803** | **0.781** | 0.783 | 0.784 | 0.782 | 0.781 |
|  | **(0.807-0.883)** | **(0.795-0.85)** | **(0.777-0.828)** | **(0.757-0.805)** | (0.763-0.803) | (0.766-0.803) | (0.764-0.8) | (0.763-0.799) |
| **HFRS+Charlson(CCI)** | 0.665 | 0.685 | 0.694 | 0.700 | 0.712 | 0.722 | 0.724 | 0.724 |
|  | (0.626-0.704) | (0.657-0.713) | (0.67-0.719) | (0.678-0.722) | (0.693-0.732) | (0.704-0.74) | (0.706-0.742) | (0.706-0.741) |
| **HFRS+CRP** | 0.706 | 0.712 | 0.722 | 0.724 | 0.730 | 0.733 | 0.733 | 0.734 |
|  | (0.648-0.764) | (0.669-0.755) | (0.685-0.759) | (0.69-0.758) | (0.701-0.758) | (0.707-0.76) | (0.707-0.759) | (0.708-0.76) |
| **2017 data (n=47431)** | | | | | | | | |
|  | **3days-mortality** | **7days-mortality** | **10days-mortality** | **14days-mortality** | **30days-mortality** | **60days-mortality** | **90days-mortality** | **6 month-mortality** |
|  | **AUROC (95%CI)** | **AUROC (95%CI)** | **AUROC (95%CI)** | **AUROC (95%CI)** | **AUROC (95%CI)** | **AUROC (95%CI)** | **AUROC (95%CI)** | **AUROC (95%CI)** |
| **HFRS** | 0.668 | 0.689 | 0.698 | 0.707 | 0.726 | 0.737 | 0.741 | 0.743 |
|  | (0.632-0.704) | (0.662-0.716) | (0.674-0.722) | (0.685-0.729) | (0.708-0.744) | (0.72-0.754) | (0.724-0.757) | (0.726-0.759) |
| **HFRS+Age** | 0.732 | 0.747 | 0.744 | 0.755 | 0.766 | 0.776 | 0.778 | 0.779 |
|  | (0.703-0.761) | (0.725-0.77) | (0.724-0.765) | (0.737-0.773) | (0.75-0.781) | (0.762-0.791) | (0.764-0.792) | (0.765-0.793) |
| **HFRS+Gender** | 0.657 | 0.690 | 0.699 | 0.703 | 0.724 | 0.736 | 0.741 | 0.742 |
|  | (0.622-0.693) | (0.663-0.717) | (0.675-0.723) | (0.682-0.725) | (0.706-0.742) | (0.719-0.753) | (0.724-0.757) | (0.726-0.759) |
| **HFRS+LDT-EWS** | 0.743 | 0.770 | 0.772 | 0.776 | **0.790** | **0.798** | **0.800** | **0.801** |
|  | (0.708-0.778) | (0.744-0.795) | (0.75-0.795) | (0.756-0.796) | **(0.774-0.807)** | **(0.783-0.813)** | **(0.785-0.815)** | **(0.786-0.816)** |
| **HFRS+NEWS** | **0.832** | **0.804** | **0.785** | **0.779** | 0.767 | 0.769 | 0.769 | 0.771 |
|  | **(0.797-0.867)** | **(0.776-0.833)** | **(0.758-0.811)** | **(0.756-0.803)** | (0.748-0.787) | (0.751-0.787) | (0.752-0.787) | (0.753-0.788) |
| **HFRS+Charlson(CCI)** | 0.668 | 0.689 | 0.698 | 0.707 | 0.726 | 0.737 | 0.741 | 0.742 |
|  | (0.632-0.704) | (0.662-0.716) | (0.674-0.722) | (0.685-0.729) | (0.708-0.744) | (0.72-0.754) | (0.724-0.757) | (0.726-0.759) |
| **HFRS+CRP** | 0.722 | 0.735 | 0.732 | 0.730 | 0.746 | 0.750 | 0.754 | 0.756 |
|  | (0.673-0.771) | (0.697-0.774) | (0.696-0.768) | (0.697-0.762) | (0.72-0.772) | (0.726-0.775) | (0.73-0.778) | (0.732-0.78) |

| **2018 data (n=50375)** | | | | | | | | |
| --- | --- | --- | --- | --- | --- | --- | --- | --- |
|  | **3days-mortality** | **7days-mortality** | **10days-mortality** | **14days-mortality** | **30days-mortality** | **60days-mortality** | **90days-mortality** | **6 month-mortality** |
|  | **AUROC (95%CI)** | **AUROC (95%CI)** | **AUROC (95%CI)** | **AUROC (95%CI)** | **AUROC (95%CI)** | **AUROC (95%CI)** | **AUROC (95%CI)** | **AUROC (95%CI)** |
| **HFRS** | 0.689 | 0.699 | 0.703 | 0.715 | 0.733 | 0.738 | 0.739 | 0.739 |
|  | (0.654-0.724) | (0.673-0.725) | (0.68-0.727) | (0.694-0.736) | (0.714-0.751) | (0.72-0.755) | (0.721-0.757) | (0.721-0.757) |
| **HFRS+Age** | 0.722 | 0.730 | 0.733 | 0.738 | 0.754 | 0.758 | 0.758 | 0.759 |
|  | (0.695-0.749) | (0.709-0.752) | (0.712-0.753) | (0.719-0.756) | (0.737-0.77) | (0.742-0.773) | (0.743-0.774) | (0.743-0.774) |
| **HFRS+Gender** | 0.690 | 0.697 | 0.699 | 0.712 | 0.730 | 0.736 | 0.738 | 0.738 |
|  | (0.655-0.725) | (0.67-0.723) | (0.674-0.724) | (0.69-0.734) | (0.71-0.749) | (0.718-0.755) | (0.72-0.756) | (0.72-0.756) |
| **HFRS+LDT-EWS** | 0.759 | 0.771 | 0.769 | 0.777 | **0.784** | **0.785** | **0.785** | **0.786** |
|  | (0.725-0.794) | (0.745-0.797) | (0.745-0.793) | (0.756-0.798) | **(0.763-0.8)** | **(0.766-0.801)** | **(0.767-0.802)** | **(0.767-0.802)** |
| **HFRS+NEWS** | **0.823** | **0.812** | **0.804** | **0.790** | 0.781 | 0.780 | 0.780 | 0.780 |
|  | **(0.791-0.856)** | **(0.787-0.836)** | **(0.781-0.827)** | **(0.768-0.812)** | (0.765-0.803) | (0.764-0.8) | (0.763-0.798) | (0.763-0.798) |
| **HFRS+Charlson(CCI)** | 0.696 | 0.710 | 0.715 | 0.725 | 0.739 | 0.744 | 0.745 | 0.745 |
|  | (0.662-0.73) | (0.684-0.737) | (0.691-0.739) | (0.703-0.746) | (0.72-0.758) | (0.726-0.762) | (0.727-0.763) | (0.727-0.763) |
| **HFRS+CRP** | 0.760 | 0.748 | 0.756 | 0.761 | 0.763 | 0.765 | 0.766 | 0.766 |
|  | (0.719-0.801) | (0.716-0.779) | (0.727-0.784) | (0.736-0.786) | (0.742-0.785) | (0.744-0.786) | (0.745-0.787) | (0.745-0.787) |
| **2019 data (n=51160)** | | | | | | | | |
|  | **3days-mortality** | **7days-mortality** | **10days-mortality** | **14days-mortality** | **30days-mortality** | **60days-mortality** | **90days-mortality** | **6 month-mortality** |
|  | **AUROC (95%CI)** | **AUROC (95%CI)** | **AUROC (95%CI)** | **AUROC (95%CI)** | **AUROC (95%CI)** | **AUROC (95%CI)** | **AUROC (95%CI)** | **AUROC (95%CI)** |
| **HFRS** | 0.659 | 0.689 | 0.702 | 0.714 | 0.737 | 0.749 | 0.751 | 0.752 |
|  | (0.626-0.692) | (0.665-0.714) | (0.68-0.723) | (0.695-0.734) | (0.72-0.754) | (0.733-0.765) | (0.735-0.767) | (0.736-0.768) |
| **HFRS+Age** | 0.737 | 0.732 | 0.731 | 0.738 | 0.755 | 0.767 | 0.768 | 0.769 |
|  | (0.709-0.765) | (0.71-0.755) | (0.711-0.751) | (0.72-0.756) | (0.74-0.771) | (0.752-0.782) | (0.754-0.783) | (0.754-0.783) |
| **HFRS+Gender** | 0.619 | 0.660 | 0.676 | 0.693 | 0.720 | 0.735 | 0.737 | 0.738 |
|  | (0.582-0.656) | (0.632-0.688) | (0.652-0.701) | (0.671-0.715) | (0.7-0.739) | (0.717-0.753) | (0.719-0.755) | (0.72-0.756) |
| **HFRS+LDT-EWS** | 0.781 | 0.783 | 0.782 | 0.786 | **0.799** | **0.799** | **0.800** | **0.801** |
|  | (0.75-0.812) | (0.779-0.825) | (0.771-0.813) | (0.77-0.808) | **(0.782-0.815)** | **(0.784-0.815)** | **(0.784-0.815)** | **(0.784-0.815)** |
| **HFRS+NEWS** | **0.800** | **0.796** | **0.788** | **0.786** | 0.774 | 0.779 | 0.781 | 0.781 |
|  | **(0.756-0.826)** | **(0.749-0.804)** | **(0.754-0.801)** | **(0.752-0.795)** | (0.756-0.792) | (0.762-0.796) | (0.764-0.798) | (0.764-0.798) |
| **HFRS+Charlson(CCI)** | 0.756 | 0.780 | 0.787 | 0.789 | 0.797 | 0.800 | 0.800 | 0.800 |
|  | (0.724-0.788) | (0.758-0.803) | (0.767-0.806) | (0.771-0.807) | (0.784-0.815) | (0.794-0.823) | (0.795-0.824) | (0.796-0.825) |
| **HFRS+CRP** | 0.664 | 0.695 | 0.702 | 0.710 | 0.731 | 0.741 | 0.743 | 0.744 |
|  | (0.623-0.705) | (0.665-0.724) | (0.676-0.727) | (0.688-0.733) | (0.712-0.75) | (0.723-0.759) | (0.725-0.761) | (0.726-0.762) |
