# Supplementary material for: Vital signs and common blood tests improve the predictive power of the Hospital Frailty Risk Score to predict poor outcomes across all adult ages
Source: PLoS One. 2026 May 5;21(5):e0348669. doi: 10.1371/journal.pone.0348669 (PMC13143055; doi:10.1371/journal.pone.0348669)
Supplement: S12 Table — (DOCX) [file pone.0348669.s012.docx]

**S12 Table: AUROC for HFRS combined with one other variable according to age groups for 8 periods of in-hospital mortality**

| **age groups <45 years (n=96963)** | | | | | | | | |
| --- | --- | --- | --- | --- | --- | --- | --- | --- |
|  | **3days-mortality** | **7days-mortality** | **10days-mortality** | **14days-mortality** | **30days-mortality** | **60days-mortality** | **90days-mortality** | **6 month-mortality** |
|  | **AUROC (95%CI)** | **AUROC (95%CI)** | **AUROC (95%CI)** | **AUROC (95%CI)** | **AUROC (95%CI)** | **AUROC (95%CI)** | **AUROC (95%CI)** | **AUROC (95%CI)** |
| **HFRS alone** | 0.692 | 0.725 | 0.744 | 0.763 | 0.762 | 0.762 | 0.762 | 0.765 |
|  | (0.592-0.792) | (0.644-0.806) | (0.669-0.82) | (0.691-0.835) | (0.698-0.826) | (0.699-0.826) | (0.699-0.826) | (0.703-0.828) |
| **HFRS +Age** | 0.729 | 0.734 | 0.732 | 0.752 | 0.771 | 0.774 | 0.773 | 0.776 |
|  | (0.625-0.834) | (0.651-0.818) | (0.654-0.81) | (0.679-0.826) | (0.707-0.834) | (0.711-0.836) | (0.711-0.836) | (0.714-0.838) |
| **HFRS +Gender** | 0.698 | 0.715 | 0.729 | 0.743 | 0.757 | 0.754 | 0.754 | 0.757 |
|  | (0.595-0.8) | (0.632-0.797) | (0.652-0.806) | (0.67-0.816) | (0.691-0.823) | (0.688-0.819) | (0.688-0.819) | (0.692-0.822) |
| **HFRS +LDT-EWS** | 0.801 | 0.831 | 0.836 | 0.848 | **0.895** | **0.880** | **0.880** | **0.877** |
|  | (0.705-0.897) | (0.761-0.901) | (0.771-0.9) | (0.788-0.908) | **(0.854-0.937)** | **(0.829-0.931)** | **(0.829-0.931)** | **(0.827-0.927)** |
| **HFRS +NEWS** | **0.899** | **0.897** | **0.898** | **0.888** | 0.864 | 0.857 | 0.857 | 0.859 |
|  | **(0.795-0.988)** | **(0.832-0.962)** | **(0.829-0.946)** | **(0.848-0.949)** | (0.813-0.916) | (0.805-0.91) | (0.805-0.91) | (0.807-0.911) |
| **HFRS +CCI** | 0.712 | 0.745 | 0.764 | 0.782 | 0.796 | 0.796 | 0.797 | 0.800 |
|  | (0.608-0.817) | (0.661-0.829) | (0.685-0.842) | (0.708-0.857) | (0.732-0.859) | (0.734-0.859) | (0.735-0.859) | (0.738-0.861) |
| **HFRS +CRP** | 0.815 | 0.862 | 0.867 | 0.875 | 0.845 | 0.837 | 0.837 | 0.837 |
|  | (0.657-0.972) | (0.769-0.955) | (0.78-0.953) | (0.797-0.953) | (0.771-0.919) | (0.763-0.91) | (0.763-0.91) | (0.763-0.91) |
| **age groups 45-64 years (n=83952)** | | | | | | | | |
|  | **3days-mortality** | **7days-mortality** | **10days-mortality** | **14days-mortality** | **30days-mortality** | **60days-mortality** | **90days-mortality** | **6 month-mortality** |
|  | **AUROC (95%CI)** | **AUROC (95%CI)** | **AUROC (95%CI)** | **AUROC (95%CI)** | **AUROC (95%CI)** | **AUROC (95%CI)** | **AUROC (95%CI)** | **AUROC (95%CI)** |
| **HFRS alone** | 0.634 | 0.663 | 0.677 | 0.685 | 0.700 | 0.705 | 0.708 | 0.709 |
|  | (0.594-0.674) | (0.632-0.694) | (0.649-0.706) | (0.659-0.712) | (0.676-0.723) | (0.682-0.728) | (0.685-0.73) | (0.687-0.732) |
| **HFRS +Age** | 0.686 | 0.693 | 0.702 | 0.704 | 0.709 | 0.715 | 0.717 | 0.717 |
|  | (0.648-0.723) | (0.662-0.724) | (0.673-0.73) | (0.677-0.73) | (0.685-0.733) | (0.693-0.738) | (0.694-0.739) | (0.694-0.739) |
| **HFRS +Gender** | 0.603 | 0.632 | 0.650 | 0.659 | 0.674 | 0.682 | 0.686 | 0.687 |
|  | (0.559-0.646) | (0.597-0.667) | (0.618-0.681) | (0.63-0.688) | (0.648-0.7) | (0.657-0.707) | (0.661-0.711) | (0.663-0.712) |
| **HFRS +LDT-EWS** | 0.772 | 0.802 | 0.809 | 0.812 | **0.824** | **0.825** | **0.827** | **0.828** |
|  | (0.737-0.808) | (0.777-0.828) | (0.786-0.831) | (0.791-0.833) | **(0.807-0.842)** | **(0.808-0.842)** | **(0.81-0.844)** | **(0.811-0.845)** |
| **HFRS +NEWS** | **0.854** | **0.847** | **0.836** | **0.821** | 0.806 | 0.809 | 0.809 | 0.809 |
|  | **(0.817-0.892)** | **(0.819-0.874)** | **(0.811-0.861)** | **(0.798-0.844)** | (0.785-0.828) | (0.789-0.829) | (0.789-0.829) | (0.79-0.829) |
| **HFRS +CCI** | 0.717 | 0.747 | 0.754 | 0.763 | 0.773 | 0.774 | 0.775 | 0.774 |
|  | (0.676-0.757) | (0.717-0.778) | (0.727-0.782) | (0.737-0.788) | (0.75-0.795) | (0.752-0.795) | (0.753-0.796) | (0.753-0.795) |
| **HFRS +CRP** | 0.725 | 0.748 | 0.754 | 0.757 | 0.769 | 0.771 | 0.772 | 0.774 |
|  | (0.677-0.774) | (0.71-0.785) | (0.72-0.788) | (0.726-0.788) | (0.743-0.796) | (0.746-0.797) | (0.747-0.797) | (0.749-0.798) |
| **age groups 65-84 years (n=131042)** | | | | | | | | |
|  | **3days-mortality** | **7days-mortality** | **10days-mortality** | **14days-mortality** | **30days-mortality** | **60days-mortality** | **90days-mortality** | **6 month-mortality** |
|  | **AUROC (95%CI)** | **AUROC (95%CI)** | **AUROC (95%CI)** | **AUROC (95%CI)** | **AUROC (95%CI)** | **AUROC (95%CI)** | **AUROC (95%CI)** | **AUROC (95%CI)** |
| **HFRS alone** | 0.568 | 0.58 | 0.589 | 0.597 | 0.617 | 0.627 | 0.629 | 0.63 |
|  | (0.548-0.587) | (0.565-0.595) | (0.576-0.603) | (0.585-0.609) | (0.606-0.628) | (0.616-0.637) | (0.619-0.639) | (0.619-0.64) |
| **HFRS +Age** | 0.557 | 0.569 | 0.578 | 0.585 | 0.604 | 0.615 | 0.616 | 0.617 |
|  | (0.537-0.578) | (0.553-0.585) | (0.564-0.593) | (0.572-0.599) | (0.592-0.616) | (0.603-0.626) | (0.605-0.627) | (0.605-0.628) |
| **HFRS +Gender** | 0.557 | 0.568 | 0.575 | 0.584 | 0.606 | 0.617 | 0.619 | 0.619 |
|  | (0.537-0.577) | (0.553-0.584) | (0.56-0.589) | (0.571-0.597) | (0.594-0.617) | (0.606-0.628) | (0.608-0.63) | (0.608-0.63) |
| **HFRS +LDT-EWS** | 0.695 | 0.711 | 0.712 | 0.724 | **0.738** | **0.736** | **0.735** | **0.735** |
|  | (0.675-0.715) | (0.696-0.726) | (0.699-0.726) | (0.712-0.735) | **(0.727-0.749)** | **(0.725-0.746)** | **(0.724-0.745)** | **(0.725-0.745)** |
| **HFRS +NEWS** | **0.797** | **0.774** | **0.764** | **0.749** | 0.729 | 0.730 | 0.731 | 0.730 |
|  | **(0.778-0.817)** | **(0.759-0.789)** | **(0.751-0.778)** | **(0.736-0.761)** | (0.718-0.739) | (0.72-0.74) | (0.721-0.741) | (0.721-0.74) |
| **HFRS +CCI** | 0.585 | 0.633 | 0.638 | 0.640 | 0.652 | 0.657 | 0.659 | 0.659 |
|  | (0.56-0.609) | (0.617-0.649) | (0.623-0.653) | (0.627-0.653) | (0.641-0.664) | (0.645-0.668) | (0.648-0.669) | (0.648-0.67) |
| **HFRS +CRP** | 0.682 | 0.690 | 0.686 | 0.689 | 0.693 | 0.693 | 0.695 | 0.696 |
|  | (0.654-0.709) | (0.669-0.71) | (0.667-0.704) | (0.672-0.706) | (0.679-0.707) | (0.679-0.706) | (0.681-0.708) | (0.682-0.709) |
| **age groups ≥85 years (n=66959)** | | | | | | | | |
|  | **3days-mortality** | **7days-mortality** | **10days-mortality** | **14days-mortality** | **30days-mortality** | **60days-mortality** | **90days-mortality** | **6 month-mortality** |
|  | **AUROC (95%CI)** | **AUROC (95%CI)** | **AUROC (95%CI)** | **AUROC (95%CI)** | **AUROC (95%CI)** | **AUROC (95%CI)** | **AUROC (95%CI)** | **AUROC (95%CI)** |
| **HFRS alone** | 0.538 | 0.513 | 0.507 | 0.507 | 0.526 | 0.539 | 0.540 | 0.540 |
|  | (0.516-0.56) | (0.496-0.53) | (0.491-0.522) | (0.492-0.521) | (0.514-0.539) | (0.526-0.551) | (0.527-0.552) | (0.528-0.553) |
| **HFRS +Age** | 0.560 | 0.546 | 0.539 | 0.541 | 0.541 | 0.549 | 0.549 | 0.549 |
|  | (0.538-0.583) | (0.528-0.564) | (0.522-0.556) | (0.525-0.556) | (0.527-0.554) | (0.536-0.562) | (0.536-0.562) | (0.536-0.562) |
| **HFRS +Gender** | 0.533 | 0.523 | 0.522 | 0.521 | 0.541 | 0.549 | 0.549 | 0.549 |
|  | (0.511-0.555) | (0.506-0.541) | (0.506-0.538) | (0.505-0.536) | (0.528-0.555) | (0.536-0.562) | (0.536-0.562) | (0.537-0.562) |
| **HFRS +LDT-EWS** | 0.676 | 0.673 | 0.672 | 0.666 | **0.710** | **0.703** | **0.703** | **0.700** |
|  | (0.654-0.698) | (0.656-0.69) | (0.656-0.687) | (0.651-0.681) | **(0.692-0.719)** | **(0.684-0.71)** | **(0.681-0.707)** | **(0.681-0.706)** |
| **HFRS +NEWS** | **0.786** | **0.756** | **0.740** | **0.724** | 0.664 | 0.663 | 0.662 | 0.662 |
|  | **(0.764-0.808)** | **(0.739-0.773)** | **(0.724-0.756)** | **(0.708-0.739)** | (0.651-0.677) | (0.651-0.675) | (0.649-0.674) | (0.649-0.674) |
| **HFRS +CCI** | 0.575 | 0.576 | 0.574 | 0.569 | 0.579 | 0.584 | 0.583 | 0.583 |
|  | (0.552-0.598) | (0.557-0.594) | (0.557-0.592) | (0.553-0.585) | (0.565-0.593) | (0.571-0.597) | (0.57-0.596) | (0.57-0.596) |
| **HFRS +CRP** | 0.685 | 0.685 | 0.685 | 0.678 | 0.672 | 0.671 | 0.669 | 0.669 |
|  | (0.656-0.713) | (0.662-0.708) | (0.664-0.706) | (0.658-0.698) | (0.655-0.689) | (0.654-0.687) | (0.653-0.686) | (0.653-0.685) |
