# Supplementary material for: Vital signs and common blood tests improve the predictive power of the Hospital Frailty Risk Score to predict poor outcomes across all adult ages
Source: PLoS One. 2026 May 5;21(5):e0348669. doi: 10.1371/journal.pone.0348669 (PMC13143055; doi:10.1371/journal.pone.0348669)
Supplement: S13 Table — (DOCX) [file pone.0348669.s013.docx]

**S13 Table: AUROC for HFRS combined with one other variable according to gender for 8 periods of in-hospital mortality**

| **Females’ data (n=207908))** | | | | | | | | |
| --- | --- | --- | --- | --- | --- | --- | --- | --- |
|  | **3days-mortality** | **7days-mortality** | **10days-mortality** | **14days-mortality** | **30days-mortality** | **60days-mortality** | **90days-mortality** | **6 month-mortality** |
|  | **AUROC (95%CI)** | **AUROC (95%CI)** | **AUROC (95%CI)** | **AUROC (95%CI)** | **AUROC (95%CI)** | **AUROC (95%CI)** | **AUROC (95%CI)** | **AUROC (95%CI)** |
| **HFRS** | 0.668 | 0.686 | 0.692 | 0.699 | 0.714 | 0.721 | 0.722 | 0.723 |
|  | (0.652-0.685) | (0.674-0.699) | (0.681-0.704) | (0.689-0.71) | (0.704-0.723) | (0.712-0.73) | (0.714-0.731) | (0.714-0.732) |
| **HFRS+Age** | 0.756 | 0.756 | 0.758 | 0.76 | 0.768 | 0.773 | 0.774 | 0.774 |
|  | (0.743-0.769) | (0.746-0.766) | (0.749-0.767) | (0.752-0.769) | (0.761-0.776) | (0.766-0.78) | (0.767-0.781) | (0.767-0.781) |
| **HFRS+LDT-EWS** | 0.777 | 0.786 | 0.789 | 0.790 | **0.795** | **0.797** | **0.797** | **0.797** |
|  | (0.761-0.793) | (0.774-0.797) | (0.779-0.8) | (0.781-0.8) | **(0.787-0.803)** | **(0.789-0.805)** | **(0.789-0.805)** | **(0.789-0.805)** |
| **HFRS+NEWS** | **0.825** | **0.805** | **0.795** | **0.786** | 0.778 | 0.777 | 0.776 | 0.776 |
|  | **(0.808-0.842)** | **(0.792-0.819)** | **(0.783-0.807)** | **(0.775-0.797)** | (0.768-0.788) | (0.768-0.787) | (0.766-0.785) | (0.766-0.785) |
| **HFRS+Charlson(CCI)** | 0.724 | 0.739 | 0.744 | 0.749 | 0.759 | 0.763 | 0.764 | 0.764 |
|  | (0.708-0.74) | (0.727-0.752) | (0.733-0.755) | (0.739-0.759) | (0.75-0.768) | (0.754-0.771) | (0.755-0.772) | (0.755-0.772) |
| **HFRS+CRP** | 0.709 | 0.719 | 0.725 | 0.729 | 0.740 | 0.742 | 0.743 | 0.743 |
|  | (0.685-0.733) | (0.702-0.737) | (0.708-0.741) | (0.714-0.744) | (0.727-0.753) | (0.73-0.755) | (0.731-0.755) | (0.731-0.755) |
| **Males’ data (n=171008)** | | | | | | | | |
|  | **3days-mortality** | **7days-mortality** | **10days-mortality** | **14days-mortality** | **30days-mortality** | **60days-mortality** | **90days-mortality** | **6 month-mortality** |
|  | **AUROC (95%CI)** | **AUROC (95%CI)** | **AUROC (95%CI)** | **AUROC (95%CI)** | **AUROC (95%CI)** | **AUROC (95%CI)** | **AUROC (95%CI)** | **AUROC (95%CI)** |
| **HFRS** | 0.626 | 0.65 | 0.664 | 0.675 | 0.696 | 0.707 | 0.708 | 0.709 |
|  | (0.607-0.645) | (0.636-0.664) | (0.651-0.677) | (0.664-0.687) | (0.687-0.706) | (0.697-0.716) | (0.699-0.718) | (0.7-0.718) |
| **HFRS+Age** | 0.708 | 0.721 | 0.724 | 0.729 | 0.743 | 0.749 | 0.750 | 0.749 |
|  | (0.692-0.723) | (0.709-0.733) | (0.714-0.735) | (0.719-0.739) | (0.734-0.751) | (0.741-0.758) | (0.742-0.758) | (0.741-0.757) |
| **HFRS+LDT-EWS** | 0.735 | 0.757 | 0.768 | 0.774 | **0.782** | **0.783** | **0.783** | **0.784** |
|  | (0.718-0.753) | (0.744-0.769) | (0.747-0.769) | (0.754-0.775) | **(0.775-0.794)** | **(0.774-0.792)** | **(0.774-0.792)** | **(0.775-0.792)** |
| **HFRS+NEWS** | **0.833** | **0.814** | **0.803** | **0.791** | 0.774 | 0.778 | 0.778 | 0.779 |
|  | **(0.816-0.851)** | **(0.801-0.827)** | **(0.791-0.815)** | **(0.779-0.802)** | (0.765-0.783) | (0.769-0.786) | (0.77-0.787) | (0.771-0.787) |
| **HFRS+Charlson(CCI)** | 0.682 | 0.700 | 0.707 | 0.714 | 0.728 | 0.735 | 0.736 | 0.736 |
|  | (0.663-0.701) | (0.686-0.714) | (0.694-0.719) | (0.702-0.725) | (0.718-0.738) | (0.725-0.744) | (0.726-0.745) | (0.727-0.745) |
| **HFRS+CRP** | 0.706 | 0.720 | 0.721 | 0.723 | 0.730 | 0.735 | 0.736 | 0.737 |
|  | (0.682-0.731) | (0.702-0.739) | (0.704-0.737) | (0.708-0.738) | (0.717-0.742) | (0.723-0.747) | (0.725-0.748) | (0.726-0.749) |
