# Supplementary material for: Vital signs and common blood tests improve the predictive power of the Hospital Frailty Risk Score to predict poor outcomes across all adult ages
Source: PLoS One. 2026 May 5;21(5):e0348669. doi: 10.1371/journal.pone.0348669 (PMC13143055; doi:10.1371/journal.pone.0348669)
Supplement: S14 Table — (DOCX) [file pone.0348669.s014.docx]

**S14 Table. AUROC for multivariate models for 8 prediction in-hospital mortality (n=378,916)**

|  | **HFRS alone** | **HFRS+NEWS** | **HFRS+LDT-EWS** | **multivariate model** |
| --- | --- | --- | --- | --- |
|  | **AUROC (95%CI)** | **AUROC (95%CI)** | **AUROC (95%CI)** | **AUROC (95%CI)** |
| 3 days-mortality | 0.648 (0.636-0.661) | **0.829 (0.817-0.841)** | 0.757 (0.745-0.769) | 0.818 (0.805-0.832) |
| 7 days-mortality | 0.669 (0.66-0.679) | **0.809 (0.8-0.819)** | 0.772 (0.763-0.78) | 0.796 (0.785-0.806) |
| 10 days-mortality | 0.679 (0.67-0.687) | **0.799 (0.79-0.807)** | 0.774 (0.767-0.782) | 0.782 (0.773-0.792) |
| 14 days-mortality | 0.688 (0.68-0.696) | **0.788 (0.78-0.796)** | 0.778 (0.771-0.785) | 0.767 (0.758-0.776) |
| 30 days-mortality | 0.705 (0.699-0.712) | 0.780 (0.773-0.787) | **0.788 (0.78-0.792)** | 0.747 (0.739-0.755) |
| 60 days-mortality | 0.714 (0.708-0.721) | 0.779 (0.773-0.786) | **0.790 (0.783-0.794)** | 0.739 (0.731-0.747) |
| 90 days-mortality | 0.716 (0.709-0.722) | 0.778 (0.772-0.785) | **0.790 (0.784-0.795)** | 0.736 (0.728-0.744) |
| 6 month-mortality | 0.716 (0.71-0.723) | 0.778 (0.772-0.785) | **0.794 (0.784-0.797)** | 0.736 (0.728-0.744) |

**Multivariate models:** HFRS+ age+ gender+ LDT-EWS+ NEWS+ CCI+ CRP

**HFRS:** Hospital frailty risk score; **NEWS:** aggregate National Early Warning Score; **LDT-EWS:** aggregate Laboratory Decision Tree Early Warning Score; **CCI:** Charlson Comorbidity Index; **CRP:** c-reactive protein test
